# The association of group IIB intron with integrons in hypersaline environments

**DOI:** 10.1186/s13100-021-00234-2

**Published:** 2021-03-01

**Authors:** Sarah Sonbol, Rania Siam

**Affiliations:** 1 Biology Department and the Graduate Program of Biotechnology, School of Sciences and Engineering, the American University in Cairo, New Cairo, Cairo, 11835 Egypt; 2 University of Medicine and Health Sciences, Basseterre, Saint Kitts and Nevis

**Keywords:** Group II introns, Integrons, CALINs, IS*200/605*, Hypersaline, Metagenomics, Mobile genetic elements

## Abstract

**Background:**

Group II introns are mobile genetic elements used as efficient gene targeting tools. They function as both ribozymes and retroelements. Group IIC introns are the only class reported so far to be associated with integrons. In order to identify group II introns linked with integrons and CALINS (cluster of *att*C sites lacking a neighboring integron integrase) within halophiles, we mined for integrons in 28 assembled metagenomes from hypersaline environments and publically available 104 halophilic genomes using Integron Finder followed by blast search for group II intron reverse transcriptases (RT)s.

**Results:**

We report the presence of different group II introns associated with integrons and integron-related sequences denoted by UHB.F1, UHB.I2, H.ha.F1 and H.ha.F2. The first two were identified within putative integrons in the metagenome of Tanatar-5 hypersaline soda lake, belonging to IIC and IIB intron classes, respectively at which the first was a truncated intron. Other truncated introns H.ha.F1 and H.ha.F2 were also detected in a CALIN within the extreme halophile *Halorhodospira halochloris*, both belonging to group IIB introns. The intron-encoded proteins (IEP) s identified within group IIB introns belonged to different classes: CL1 class in UHB.I2 and bacterial class E in H.ha.Fa1 and H.ha.F2. A newly identified insertion sequence (IS*Hahl1*) of IS*200/605* superfamily was also identified adjacent to *H. halochloris* CALIN. Finally, an abundance of toxin-antitoxin (TA) systems was observed within the identified integrons.

**Conclusion:**

So far, this is the first investigation of group II introns within integrons in halophilic genomes and metagenomes from hypersaline environments. We report the presence of group IIB introns associated with integrons or CALINs. This study provides the basis for understanding the role of group IIB introns in the evolution of halophiles and their potential biotechnological role.

**Supplementary Information:**

The online version contains supplementary material available at 10.1186/s13100-021-00234-2.

## Background

**Fig. 1:**
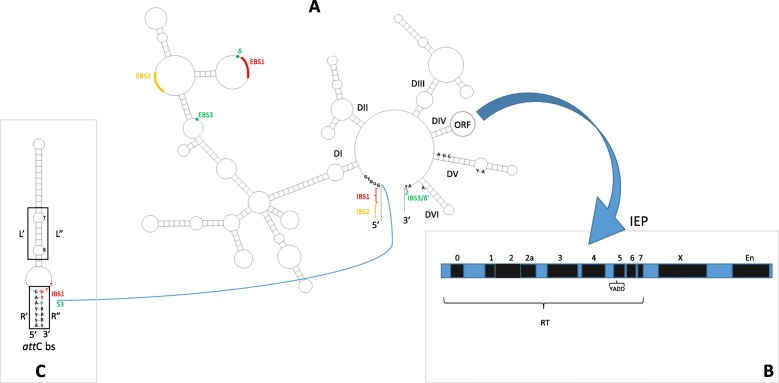
General secondary structure of group II intron RNA, *att*C site and domains of IEP. Group II intron is composed of 6 domains (DI-DVI) at which DI and DV form its catalytic core (**a**). Intron encoded protein (IEP) is encoded by ORF in DIV (A). The main domains of an IEP ORF (RT: reverse transcriptase, X: maturase and En: endonuclease are depicted in the schematic diagram of IEP (**b**). Recognition of target site occurs mainly via base-pairing between short sequences at 5′ exon (Intron binding sites IBS1 and 2) with exon binding sites (EBS1 and 2) on the intron and either IBS3 or δ’ on exon 3′ (based on intron class) with EBS3 or δ on the intron (**a**). In case of group IIC, IBS2 is replaced by a hairpin structure such as *att*C bottom strand (bs) in group IIC-*att*C (**c**) at which the intron is inserted at the R” sequence into the consensus sequence TTGT/T (IBS1/IBS3)

Group II introns are mobile genetic elements (MGE) s with catalytic RNA (ribozyme) and retroelements properties [1, 2], linked to non-Long terminal repeat elements [3, 4]. They are found in mitochondrial and chloroplast genomes of lower eukaryotes and plants, and in known bacterial and archaeal genomes [1, 3]. The transcribed ribozyme catalyzes the excision of the intron and its integration into new locations with the aid of an intron-encoded protein (IEP) [3]. Although the RNA sequence of the ribozyme is poorly conserved [2], it can be classified into 3 major groups (IIA, IIB and IIC) [5]. Group II introns classification is based on its conserved secondary and tertiary structure where it forms 6 double helical domains (DI-DVI) radiating from a central wheel [3, 5] (Fig. 1a). DI and DV form the minimal catalytic core of the ribozyme, while DIV encodes the intron open reading frame (ORF) [5]. Catalysis is promoted by the binding of Mg^2+^ ions to AGC triad [6] (CGC in case of group IIC introns [7]) and to an AY bulge, located in DV [6] (Fig. 1a). Amongst the 6 double helical domains, only DV and DVI are highly conserved [2].

Additionally, group II introns can be classified into subgroups, mitochondrial-like (ML), chloroplast-like class I (CL1), chloroplast-like class II (CL2) and bacterial classes A-E, based on their IEP [8]. Bacterial group II introns contain all previously mentioned subgroups, whereas organelles contain only CL and ML subgroups [9]. The IEP can function as a reverse transcriptase (RT 0–7 subdomains), a maturase (X domain) which binds to the intron RNA to stabilize the secondary structure and assist RNA splicing, and a DNA endonuclease (En domain) [4, 5, 8]. A “YADD” motif necessary for the reverse transcription activity is highly conserved in all bacterial IEPs within RT5 domain [4, 6] (Fig. 1b). Each IEP subgroup can be associated with one RNA subclasses including mitochondrial-like (IIA1), chloroplast-like class I (IIB1), chloroplast-like class II (IIB2), bacterial class A (IIA/B), bacterial class B (IIB-like), bacterial class C (IIC), bacterial class D (IIB-like) and bacterial class E (IIA/B) [5]. Most bacterial IEPs are found within MGEs such as plasmids or insertion sequences (IS) [4].

Mobilization of group II introns occurs through an RNA intermediate leading to their duplication [10]. The ribozyme in its conserved secondary structure can catalyze its own splicing (excision) from a precursor transcript [11]. Intron splicing usually occurs via 2 sequential transesterification steps [5] starting with a nucleophilic attack of the hydroxyl group in a DVI conserved bulged adenosine (branching pathway) and ending with the formation of an intron lariat and the ligation of the 5′ and 3′ exons [1, 12]. A less efficient splicing mechanism may occur by water hydrolysis, without the aid of the bulged “A”, resulting in a linear excised intron rather than a lariat [12]. The hydrolysis pathway seems to be more common in group IIC introns [11]. The excised intron transcript (RNA) remains associated with the IEP forming a ribonucleoparticle (RNP), which is then inserted (reverse splicing) into either an intronless allele (retrohoming) or to a non-cognate site (retrotransposition or ectopic transposition) with a lower frequency [1]. Reverse splicing into dsDNA requires cleavage of the sense strand, where the intron transcript gets inserted, followed by a cleavage in the antisense strand catalyzed by the En domain of IEP. En-independent retrohoming is connected to DNA replication since single stranded DNA (ssDNA) stretches are formed eliminating the need for a second strand cleavage [1]. Yet, reverse splicing into ds or ssDNA independent of DNA replication can also occur but less frequently [1]. Various studies have shown that putative intron boundaries have a consensus sequence of “GUGYG” at the 5′ end and “AXX(X)XRAY” at the 3′ end, including the bulged “A” in DVI [7]. To be inserted, the IEP recognizes specific nucleotides in the exons flanking the target site, followed by base pairing between short sequences in the DI loop of the intron RNA (Exon Binding Sites EBS) and sequences in the target site (Intron Binding Sites IBS) [1, 13]. In group II A and B, 5′ exon is recognized mainly by 2 base pairing interactions; IBS1-EBS1 (6 bp) and IBS2-EBS2 (6 bp) [11]. In case of 3′ exon, its first 1–3 nucleotides (δ’) pair with (δ) position upstream of EBS1 in group IIA introns, while in group IIB, the first nucleotide of 3’exon (IBS3) pairs with (EBS3) position in DI double helix of the intron [1]. On the other hand, group IIC introns exhibit some variations in their target site recognition; both IBS1-EBS1 and IBS3-EBS3 interactions can be identified. However, pairing in IBS1-EBS1 is 3–4 bp rather than 6 bp. Up to this point there’s no evidence for a IBS2-EBS2 interaction. It was identified that a stem-loop of a Rho-independent terminator or other inverted repeat structure such as an *att*C site is located upstream of IBS1 [1, 8].

*att*C sites are recombination sites usually found at the 3′ end of an integron gene cassette, which can be recognized by the integron integrase (IntI), leading to integration or excision of integron gene cassettes [14]. An *att*C site is composed of 4 successive binding sites denoted by R”, L”, L’ and R’. R” and R’ are the only conserved domains with the consensus of 5′-RYYYACC-3′ and 5′-GTTRRRY-3′, respectively [14, 15]. The recombination reaction only involves the *att*C bottom strand (bs) which forms a stem loop structure, where R” and L” pair with R’ and L’ forming the R and L boxes, respectively [16] (Fig. 1c). Group IIC-*att*C introns form a specific lineage of group IIC introns, this was found to be inserted directly after or into the stem-loop motif of the *att*C site bs, in an opposite orientation to the gene cassettes transcription [8, 17]. Group IIC-*att*C introns can also integrate into *att*C sites within clusters of *att*C sites lacking a neighboring integron-integrase [17] (CALINs) [18]. The majority of these introns were inserted into a consensus sequence of TTGT/T (IBS1/IBS3) within an *att*C site [8, 17] (Fig. 1c). Moreover, despite *att*C sites preference, these introns were found to retain their ability to target other putative transcriptional terminators. This led to the suggestion that group IIC-*att*C introns might be involved in integron gene cassette formation by separately targeting an isolated *att*C site and a transcriptional terminator of any gene, followed by joining this *att*C site to that gene by homologous recombination [8]. Thus, presence of Group IIC-*att*C introns within gene cassette arrays may represent an intermediate step in the formation of some gene cassettes [8].

Members of group IIA and IIB introns have been successfully utilized as gene targeting vectors (targetrons) with high integration efficiency and target specificity [19]. On the other hand, group IIC introns have never been used in such applications, as their reverse splicing mechanism is not fully understood [19]. Furthermore, IEPs have a high potential to be used as RTs in different biotechnological applications that involve cDNA synthesis such as qRT-PCR and RNA sequencing (RNA-seq). Their high fidelity and lack of RNase H activity enables their reuse of RNA templates, making them superior to commercially available RTs [19].

In this study, we investigated group II introns associated with integrons and CALINs in 28 previously assembled metagenomes (1,236,831,758 nucleotides and 658,054 contigs) from different hypersaline environments and all publically available halophilic genomes (104 genomes). We identified -for the first time- group II introns belonging to different classes within integron gene cassette arrays in the metagenome from the hypersaline Tanatar-5 Soda lake, Russia (IIB/CL1 and IIC-*att*C) and within the genome of the extreme halophile *Halorhodospira halochloris* DSM 1059 (IIB/E)*.* Tanatar-5 soda lake is an alkaline hypersaline lake with a pH of 9.9 and a salinity of 170 gl^− 1^ [20] with a highly active microbial sulfide cycle [21]. *H. halochloris* is an obligate anaerobic phototroph that inhabits environments of highly saline, and alkaline conditions. The optimal growth conditions of *H. halochloris* requires the presence of sulfide, a pH of 8.1–9.1 [22] and salt concentration of 140–270 gl^− 1^ [22]. Furthermore, we have identified a new IS element (IS*Hahl1*) of IS*200/605* superfamily adjacent to the CALIN sequence in *H. halochloris,* and submitted the sequence to the ISfinder database [23]. We investigate putative links between such mobile genetic elements, in the halophile genome and metagenome from a hypersaline environment, and whether an essential synchronized mobilization events occur enabling the adaptation of halophiles in salty environments.

## Results

### Different intron encoded protein (IEP) classes associated with hypersaline integrons and CALINS

We mined 658,054 contigs (1,236,831,758 bp) from 28 hypersaline aquatic metagenomes for integrons and identified CALINs, rather than full integrons, in most sites (unpublished results). Annotation of the identified gene cassettes revealed the presence of two-group II intron RT/maturases in 2 different contigs (LFIK01005867 and LFIK01005957) from Tanatar-5 hypersaline Soda Lake (TSL) in Kulunda steppe in Siberia, Russia. Here we refer to them as TSL1 and TSL2, respectively. The identified group II introns in TSL1 and TSL2, at which the first was truncated, were referred to as uncultured halophilic bacterium introns 1 and 2 (UHB.F1 and UHB.I2), respectively. On the other hand, no group II RTs were found within integrons or CALINS of the examined 1,444,498 contigs (1,750,281,271 bp) from the 22 marine or 7 freshwater previously assembled metagenomes.

As TSL1 and TSL2 contigs, with group II introns, were identified from a hypersaline lake, it is expected that they belong to halophilic microorganisms. Thus, we examined publically available complete and partial 104 halophilic genomes to get a clearer picture of the group II introns associated with integrons in halophiles. Only 2 group II intron RT/maturases, in the same CALIN, in the genome of the extreme alkaliphilic and halophilic purple sulfur gammaproteobacterium *Halorhodospira halochloris* DSM 1059 [22] were detected. Apart from the identified CALIN, only one other group II intron RT was detected in *H. halochloris* (previously reported in NCBI nr database with the accession number WP_096410353.1). Fragmented introns identified within *H. halochloris* CALIN were denoted by H.ha.F1 and H.ha.F2.

**Fig. 2:**
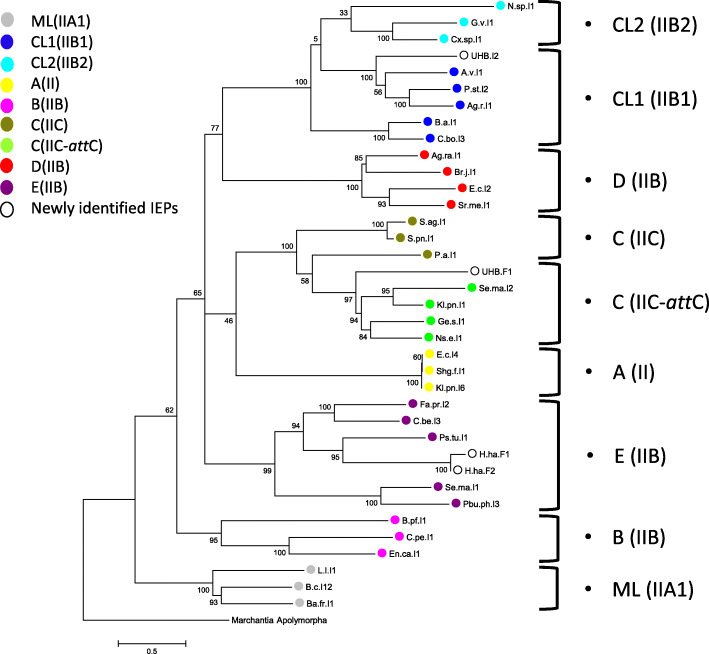
Phylogenetic tree of identified putative IEPs with IEPs from different bacterial groups. UHB.F1 clusters with group IIC-attC, UHB.I2 with group IIB (Chloroplast-like1 class) and both H.ha.F1 and H.ha.F2 with group IIB (bacterial class E). IEPs abbreviations are based on their introns nomenclature in group II introns database [25, 26]. Mitochondrial IEP from Liverwart *Marchantia polymorpha* is used as an outgroup. Bootstrap values are indicated as percentages of 1000 replicates

To assign UHB.F1, UHBI2, H.ha.F1 and H.ha.F2 to specific intron classes, we constructed maximum likelihood phylogenetic tree with different classes of bacterial IEPs (Fig. 2). The phylogenetic tree revealed that UHB.F1 belongs to Group IIC-*att*C class, known to be associated with integrons [8, 17, 24]. On the other hand, UHB.I2 clustered with class CL1(IIB1), whereas H.ha.F1 and H.ha.F2 clustered with bacterial class E (IIB). Blastx analysis of the identified IEPs nucleotide sequences against group II intron database [25, 26] confirmed the results of our phylogenetic analysis. The closest hit to UHB.F1 was Ge.s.I1 of group IIC-*att*C class from *Geobacter sulfurreducens* with 50% identity and 62% similarity. Since the group II intron database is limited in number of sequences, we blasted the sequence against the vast NCBI nr database, better hits were obtained, as the best hit was a group II intron RT from a *Verrucomicrobia* bacterium (sequence ID: NBB81160.1) with 80% identity and 87% similarity. However, the X domain of the IEP was detected in 3 different frames due to a small indel and an 11 bp insertion at the C-terminus. In addition, we were not able to locate the exact start of the translated protein as the predicted start by Prodigal [27] in Integron Finder tool detected the start at c (3899) missing few amino acids upstream that are actually part of the RT0 domain (Additional file 1 Fig. S1 and S2). The whole RT0 domain was still incomplete missing few upstream amino acids indicating a possible deletion (Additional file 1 Fig. S1). In case of UHB.I2, the closest hit was Sh.sp. I2 (CL1/IIB1) from a *Shewanella* sp., with 53% identity and 69% similarity, when blasted against group II introns database, whereas its closest hit on NCBI was a group II intron RT from *Legionella birminghamensis* (sequence ID: WP_054523790.1) with 56% identity and 70% similarity.

Multiple sequence alignment of UHB.F1 and UHB.I2, each with its closely related IEPs showed all required domains for IEPs lacking the endonuclease domain (En^−^) (Additional file 1 Fig. S1 and S3).

The aligned part of H.ha.F1 and H.ha.F2, which covers 60% of H.ha.F1 C-terminus, showed 95% identity to each other, with Ps.tu. I1 (E/IIB) from *Pseudoalteromonas tunicata* being their closest homolog. Both H.ha.F1 and H.ha.F2 showed 70% similarity to Ps.tu. I1 (E/IIB). H.ha.F1 and H.ha.F2 had also shown 63.1–64.3% similarities to IEPs from one uncultured archaeon ANME-1 (UA.I6, UA.I7 and UA.I8). In case of H.ha. F1, an internal stop codon and a 79 bp-deletion were identified which most likely led to a frameshift and loss of RT3 and RT4 domains; whereas in H.ha. F2, the N-terminus, with domains RT0–4 necessary for the RT function, was absent (Additional file 1 Fig. S2 and S4).

### Group II intron RT/maturases from Tanatar-5 hypersaline soda Lake (TSL1) harbors a truncated group IIC-*att*C intron within an array of gene cassettes

**Fig. 3:**
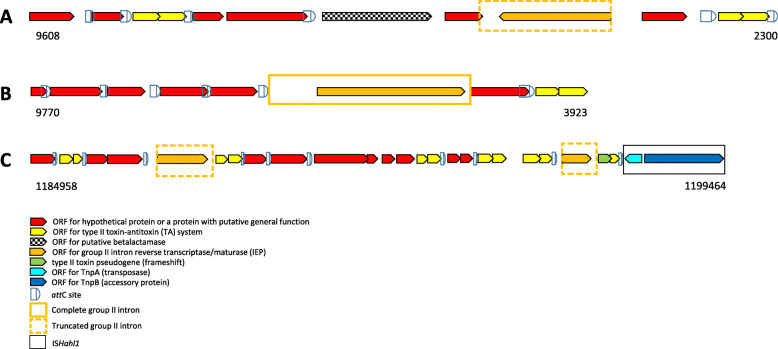
Schematic representation of identified gene cassette arrays where group II introns are inserted. **a** TSL1 (LFIK01005867; c (2300..9608)), **b** TSL2 (LFIK01005957;c (3923..9770)), **c** *H. halochloris* CALIN (NZ_AP017372.2; 1,184,958..1199464). Arrow heads of different ORFs show the direction of their transcription. Colored legend show different genetic elements depicted. Map coordinates are indicated below each schematic representation

In order to identify group II introns to which the identified IEPs belong, sequences flanking these IEPs were further analyzed. In case of TSL1, a truncated group IIC-*att*C intron (UHB.F1) was detected (Additional file 1 Fig. S5) within an array of gene cassettes. The intron was inserted in an opposite orientation to the adjacent gene cassettes. Being at the periphery of the TSL1 contig (9835 bp), the 5′ region of the detected gene cassette array seems to be missing. Thus, it is not clear whether it is a CALIN or part of a full integron with essential integron components at the 5′ region such as *int*I gene, *att*I and P_C_ promoter (Fig. 3a and Additional file 2 Table S1).

We identified the 3′ end of the intron which showed typical folding of DV and DVI loops (Additional file 1 Fig. S6). However, although all RT domains were detected in the identified IEP, the RT0 domain missed few amino acids indicating a deletion at its N terminus. It is more likely that the identified intron is a 5′ truncated intron as it was challenging to find a proper start or a properly full folded intron.

The gene cassette at which the intron is inserted has 3 other ORFs, 2 encode for conserved hypothetical proteins, while the first ORF encodes for a putative serine hydrolase (betalactamase transpeptidase). Two other gene cassettes within TSL1 encode for type II toxin-antitoxin (TA) systems. Other ORFs within the array either encode for conserved hypothetical proteins or show no similarities with proteins in nr database (Fig. 3a and Additional file 2 Table S1).

Since previous studies showed that internal promoters within the oppositely inserted introns can drive the expression of gene cassette ORFs at the 3′ end of the array (those present after the intron) [3], we searched for the presence of putative promoters within UHB.F1 and its upstream region that could drive the transcription of gene cassettes at the 3′ end of the gene cassette array and upstream of the intron. Four potential promoters were detected (Additional file 1 Fig. S5 and Additional file 2 Table S1). Perhaps one or more of these putative promoters is responsible for the expression of just one downstream ORF encoding for a hypothetical protein, since the TA operon in the next gene cassette had two predicted promoters in addition to two more predicted promoters within the antitoxin gene that could drive the transcription of the toxin gene (Additional file 2 Table S1).

### TSL2 and a CALIN within *Halorhodospira halochloris* genome harbor group IIB introns

Following the same steps described for the identification of UHB.F1 in TSL1, we examined the sequences surrounding the detected group II intron RT in TSL2 and within *H. halochloris* CALIN. Unexpectedly, we identified group IIB introns associated with gene cassette arrays in TSL2 and in the genome of *H. halochloris*. In TSL2 (9772 bp contig), a full group IIB1 intron was detected, with its IEP belonging to CL1 class (Fig. 3b, Additional file 1 Fig. S2 and S5, and Additional file 2 Table S1). Unfortunately, the array was at the periphery of the contig, as with the TSL1 contig. Thus, the 5′ region of the integron or the CALIN was missed and the identified ORF in the first gene cassettes was relatively short (144 bp) with no start codon (Fig. 3b and Additional file 2 Table S1).

**Fig. 4:**
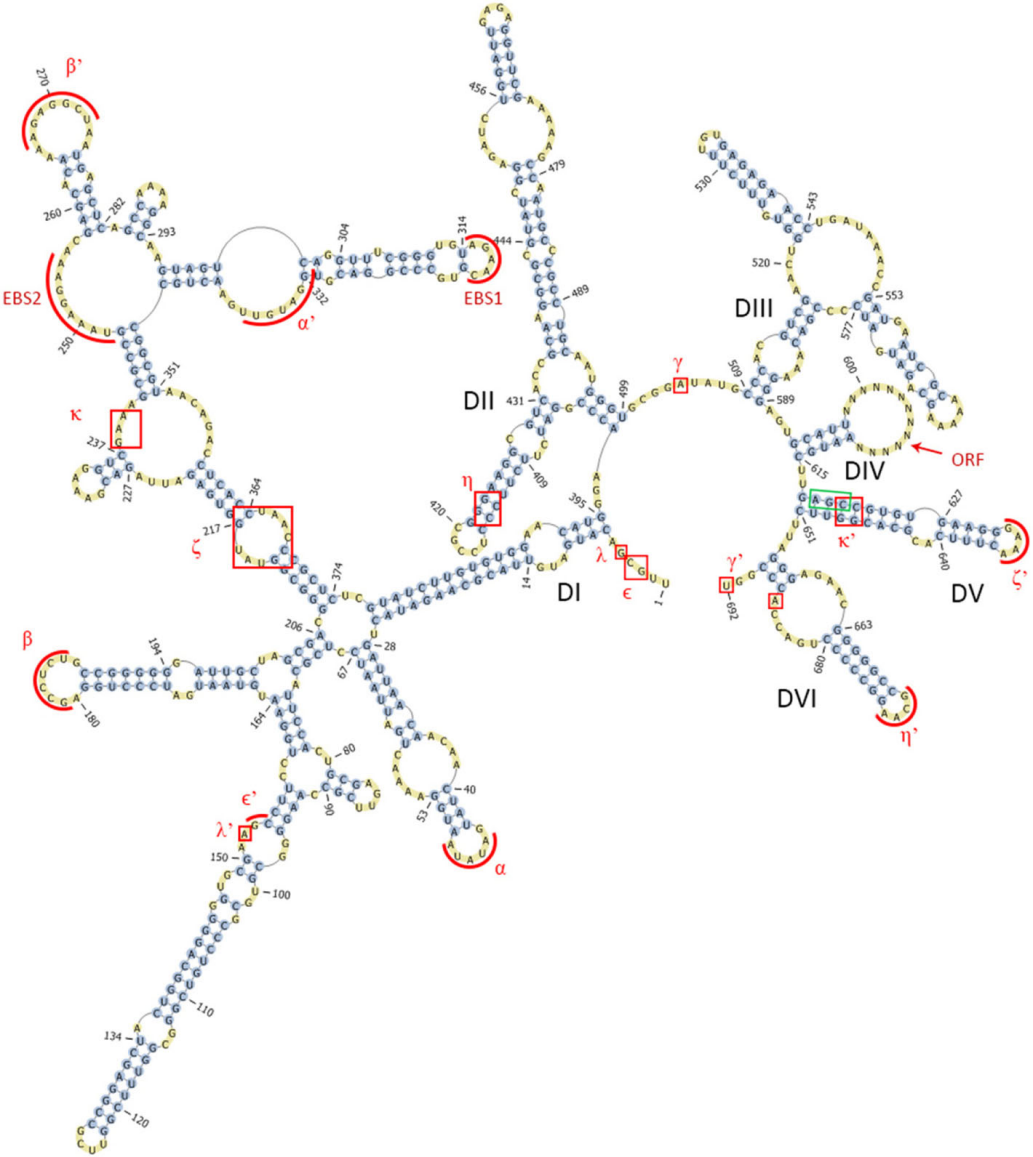
Secondary structure of group II intron UHB.I2. UHB.I2 identified in TSL2 contig shows necessary sequences required for intron splicing and reverse splicing. Important sequences are shown within red rectangles or curved lines. EBS1 and EBS3 are important for base-pairing with target site in flanking exons, whereas other identified sequences are necessary for intron folding (Watson-Crick α-α’, β-β’, Ɛ-Ɛ’and γ-γ’ and non-Watson-Crick ĸ-ĸ’ and λ-λ’ internal base-pairing and tetraloop-receptor interactions ζ-ζ’ and η-η’). Conserved catalytic “AGC” triad in DV is shown in a green rectangle

**Fig. 5:**
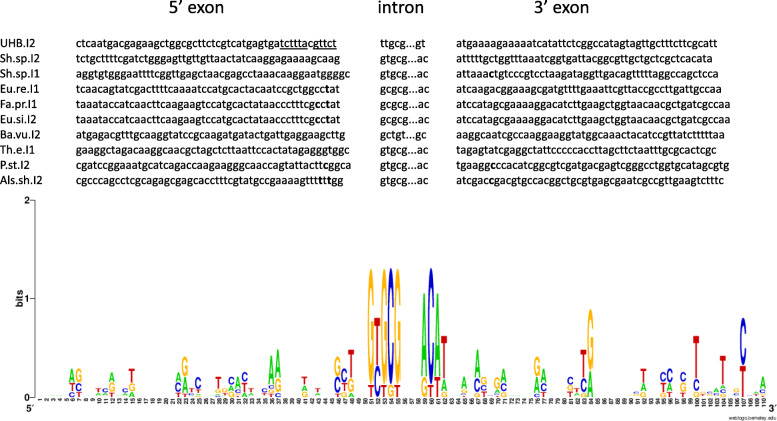
UHB.I2 flanking exons logos with closer hits. The logo shows a conservation in the target site. IBS1 and IBS2 sequences in UHB.I2 5′ exon are underlined

The secondary structure of the intron showed a typical IIB intron with essential sequences required for intron folding and base pairing with target site, except for IBS3-EBS3. EBS3 base exists within a bulge at the folded structure [1]; however, the anticipated bulge was absent (Fig. 4). The intron boundaries were different from the known consensus sequence 5′-GUGYG..AY-3′, as the boundaries in this case were 5′-UUGCG..GU-3′. Unlike group IIC-*att*C introns, UHB.I2 was inserted in the same orientation of the gene cassettes in the array. Several promoters were predicted within UHB.I2 that could serve as promoters for the IEP ORF or downstream ORFs in the array (Additional file 1 Fig. S5 and Additional file 2 Table S1). Although upstream stem-loop structures were only reported within group IIC introns, we detected UHB.I2 intron immediately after an *att*C site in the array (Additional file 1 Fig. S7). Examination of UHB.I2 flanking exons with homologous introns showed poor conservation for both exons except for the first 2 nucleotides in 3′ exon (Fig. 5).

*H. halochloris* introns identified within its CALIN (H.ha.F1 and H.ha.F2) were both fragmented at their 5′ end, and we only identified their 3′ end of the intron (DV and DVI) and part of the IEP ORFs (Additional file 1 Fig. S2 and S5). Folding of DV and DVI, depicting the 3′ part of a group IIB intron were predicted in both intron RNAs (Additional file 1 Fig. S6). Here again, putative promoters were predicted within H.ha.F1 and H.ha.F2 (Additional file 1 Fig. S5 and Additional file 2 Table S1). In all cases putative promoters directly upstream of all identified introns were detected (Additional file 2 Table S1).

### Gene cassette arrays with identified group II introns are all associated with type II toxin-antitoxin (TA) systems

Following the identification of group II introns within integrons and CALINs, we analyzed detected ORFs within these integrons. All ORFs were BLASTed and annotated. We found that the three examined arrays contain type II TA systems of various types (Fig. 3 and Additional file 2 Table S1). Two TA system gene cassettes within TSL1 array were detected. In case of *H. halochloris* CALIN, most of the ORFs identified within the gene cassettes belonged to toxins and antitoxins of type II TA systems giving rise to five TA systems within the CALIN. Three of the five were of the same type (BrnT/A family). Both H.ha.F1 and H.ha.F2 were inserted within gene cassettes with TA operons. However, in the gene cassette at which H.ha.F2 is inserted, a frameshift within the *HicA* toxin gene was found, casting doubt on its possible expression. In case of TSL2, the TA system identified was just downstream of the last *att*C site in the array. Upstream to all identified TA operons, putative promoters also existed.

### An insertion sequence (IS*200/605)* lies directly downstream of *H. halochloris* CALIN

The relatively small length of TSL1 and TSL2 contigs limited our ability to search for *int*I genes or other MGEs close to the identified gene cassette arrays. This was not an obstacle in case of *H. halochloris* due to the availability of its full genome sequence. Examination of *H. halochloris* genome revealed the presence of just one CALIN with absence of *int*I genes in the whole genome. This CALIN contained 10 gene cassettes, with 6 ORFs in one gene cassette (detailed annotations in Additional file 2 Table S1). Directly, downstream of the last *att*C in the identified CALIN, we found a new insertion sequence (IS) (Fig. 3c), that we submitted to the ISfinder database [23] under the name IS*Hahl1*. It belonged to the complex IS*200/605* family that has no inverted repeats. Instead, palindromic hairpin structures were identified at both ends. Such structures are known to be involved in transposition [28]. The hairpin structures were compared to that of IS*CARN6*, the closest homologue in ISfinder database showing 66% identity to IS*Hahl1* (Additional file 1 Fig. S9).

Two ORFs of opposite orientations were identified within IS*Hahl1*; *tnp*A and *tnp*B. The former (80% identity to IS*CARN6* TnpA) encodes for a putative HUH enzymes superfamily transposase, whereas the latter (56% identity to IS*CARN6* TnpB) encodes for an accessory protein that is speculated to be involved in negative regulation of transposition [28]. The configuration of the two ORFs is characteristic of IS*605* group within the IS*200/605* family [28]. With the aid of ISEScan pipeline [29], An isoform of IS*Hahl*1 was found with 98% identity (Additional file 2 Table S1) about 70 kb downstream. Several other IS*605* group elements within the genome were identified; however, they were either partial, with frameshifts or missing parts in *tnp*A and *tnp*B genes, most probably rendering them nonfunctional (Additional file 2 Table S1).

To determine if the studied genetic elements in *H. halochloris* are transcribed from leading or lagging strands, we searched for the origin of replication (*Ori*C). GammaBOriS tool [30] results showed that the most probable *Ori*C position lies between 2,787,842–2,789,091 bp in the 2,834,560-bp-*H.haochloris*-genome. Based on this position, the top strand of the gene cassettes in the identified CALIN seems to be transcribed on the leading strand. This also means that H.ha.F1, H.ha.F2, IS*Hahl1* and its isoform are on the leading strand, while the *att*C sites’ bottom strands in the CALIN are on the lagging strand.

## Discussion

### Identification of integron-associated group II introns sequences from a hypersaline metagenome and in *H. halochloris*

Presence of group II introns has been reported in different bacterial, archaeal and organeller genomes [1]; however, their association with integrons has been limited to group IIC introns [8, 17, 24, 31], and in most reported cases, the association was confined to IIC-*att*C subclass [8, 17, 24]. To date, none of these integron-associated-introns have been found in halophiles. Here, we have analyzed group II introns associated with integrons and CALINs in publically 104 available halophilic genomes and previously assembled 28 hypersaline metagenomes (a total of 658,054 contigs corresponding to 1,236,831,758 bp) in an attempt to understand the role of specific mobile genetic elements in environmental adaptation of halophiles. We have detected integron-associated-group II introns, class IIC-*att*C and class IIB in the metagenome of the hypersaline Tanatar-5 Soda lake, in Russia and in the genome of the extreme halophile *Halorhodospira halochloris.* Intriguingly, we did not find any group II introns associated with integrons in the remaining analyzed metagenomes. However, we cannot rule out the probability of detecting integron-associated-group II introns in other hypersaline metagenomes. Our findings infer an adaptation role for these integrons in hypersaline alkaline environments. Group II introns have high biotechnological potential, where few members belonging to IIA and IIB classes, have already been commercialized as targetrons [19].

Our newly detected group IIC-*att*C intron, UHB.F1, from the metagenome of the hypersaline Tanatar-5 lake in Russia, is inserted in opposite orientation to the transcription of the adjacent gene cassettes, which is typical of group IIC-*att*C introns [8, 17, 24]. However, it was a 5′ truncated intron with frameshifts near its 3′ end. On the other hand, UHB.I2, isolated from the same metagenome, belonged to group IIB1 rather than group IIC and its IEP clustered with CL1 class. This intron was in the same orientation of the gene cassettes transcription, just downstream an *att*C site. In one reported case in an integron in *Enterobacter cloacae*, an unusual group IIC intron (not a IIC-*att*C) was at the same orientation of adjacent gene cassettes, as it was inserted within the top strand of an *att*C site rather than the usual bs target site [31]. Unlike other group II introns, group IIC introns possess a stem-loop structure upstream of the insertion site [1, 8]. *att*C bs seems to serve the function of the upstream stem-loop, in group IIC-*att*C, as known IIC-*att*C introns are inserted within putative *att*C bottom strands [8]. Although in case of group IIB introns, no role of upstream secondary structures has ever been reported, it is intriguing to speculate a role of the secondary structure in the identification of target site, as the *att*C top strand can also form a non-recombinogenic hairpin.

Upon examination of the flanking exons of UHB.I2, there was no sequence conservation in its flanking exons. However, it showed an AT rich 3′ exon (Fig. 5). The same observation was found with *Lactococcus lactis* Ll.LtrB intron (group IIA), where reverse splicing was inhibited by increasing the exon’s GC content [13]. Further experiments should be performed to determine the role of the UHB.I2 AT rich 3′ exon in reverse splicing. UHB.I2 intron seems to fold into nearly typical group IIB intron secondary structure yet the bulge containing the EBS3 site in the DI coordination loop, was missing (Fig. 4). It is likely that IBS3 on the target site interacts with an alternative EBS3 site or position.

### Identification of putatively essential upstream secondary structures for group II intron mobilization in *H. halochloris*

All our identified IEPs lacked an endonuclease domain (En^−^), which is in more than half the bacterial group II introns IEPs [1, 5]. Since En^−^ IEPs are incapable of a second strand cleavage, they depend on the host replication machinery for insertion into new target sites [1].

Based on GammaBOriS [30] identification of the origin of replication in *H. halochloris*, H.ha.F1 and H.ha.F2 are inserted within the leading strand rather than the lagging strand; a documented yet rare phenomenon [1]. Furthermore, despite the above mentioned reliance of En^−^ IEPs group II introns on host replication machinery for complete retrohoming and retrotransposition, a possible minor retrohoming pathway independent of DNA replication can exist, at which introns can reverse splice into double stranded (ds) or transiently ssDNA target sites [1].

In *att*C recombination, replication is not only important for the formation of the folded bs, but also for the resolution of recombination products [32, 33]. However, the presence of single stranded proteins (SSP) hampers the formation of a fully folded *att*C bs in absence of integron integrase (IntI) [34, 35]. In the absence of IntI, an equilibrium between the opened *att*C bs and a partially structured *att*C bs which forms a complex with SSPs exists [35]. We did not detect *intI* genes in the genome of *H. halochloris,* despite the presence of a CALIN*.* Therefore, the role of these gene cassettes in the absence of *int*I in the genome of *H. halochloris* raises a question of whether they function just as reservoirs for horizontal transfer of gene cassettes or they have an unidentified role. The identified introns within *H. halochloris* CALIN, H.ha.F1 and H.ha.F2 are both 5′ truncated introns and only their 3′ ends were identified, and important RT domains within their IEP ORFs were also absent most probably leading to non-functional IEPs. It is already documented that fragmented introns with frequent frameshifts are more commonly found than full-length introns in bacterial genomes [9]. Yet, a putatively functional IEP ORF (80% identical to H.ha.F1 IEP) was detected, about 6.5 kb upstream of the CALIN (Acc.no WP_096410353.1). Perhaps both H.ha.F1 and H.ha.F2 were formed as a result of incomplete reverse transcription due to replication slippage caused by the presence of hairpin structures. Manually and with the aid of MFOLD [36], we have detected an *att*C-like structure upstream of H.ha.F1 (Additional file 1 Fig. S10A) and a putative *att*C site upstream of H.ha.F2, showing a nearly typical *att*C site bs secondary structure (Additional file 1 Fig. S10B). Again, the presence of these secondary structures before group IIB introns further suggests their possible role in recognition of target sites.

### Clustering of MGEs requiring ssDNA in hypersaline group II introns

Coexistence of group II introns, integrons and IS elements may have a combined role in increasing genomic plasticity in extreme hypersaline environments. In *H. halochloris* CALIN, we have identified directly downstream of the last gene cassette, at which H.ha.F2 is inserted, a new IS element “IS*Hahl1*”. IS*Hahl*1 belongs to IS*605* group of IS*200/605* family were t*np*A and *tnp*B are transcribed in opposite directions.

Insertion sequences belonging to IS*200/605* family are distinguished from other IS elements by their transposition mechanism; 1- utilizing obligatory ssDNA intermediates, 2- absence of nucleotides loss or gain, 3- requiring transposase “TnpA” belonging to the “HUH” superfamily of enzymes rather than the “DDE” family of classical IS elements [28, 37] and 4- the presence of hairpin structures at both ends [28]. Transposition is strand specific and follows a “peel and paste” mechanism at which an excised circular single stranded intermediate integrates into a single stranded target site [28]. For transposition to take place, both ends need to be single stranded at the same time. Thus, a link between IS*200/605* family members’ transposition and replication was reported, with more frequent transposition into the lagging strand [28]. Unexpectedly, the IS active “top” strand that carries the target sequence was found on the leading strand, yet *tnp*A gene was transcribed on the lagging strand. In some cases, presence of IS*200/605* elements on the leading strand was attributed to genomic rearrangements [28]. In fact, it was suggested that identical IS*605* elements in *H. pylori* had caused rearrangement within its genome [38]. The presence of an isoform to IS*Hahl*1 (98% identity) and other IS*605* elements with high homology to IS*Hahl*1 (Additional file 2 Table S1) may allow such rearrangements to occur by homologous recombination. The rationale behind our mining for similar IS element was to inspect the possibility of mobilization of the adjacent CALIN sequence. Yet the large distance between the nearest homologous upstream IS605 element at 460538–461995 bp (¬735 kb) confines this possibility. Even though previous studies reported a link between IS*200/605* transposition and replication, high transposition frequencies were reported with DNA repair mechanisms when large ssDNA stretches become available [39]. Moreover, it is worth noticing that IS*200/605* elements belong to HUH endonuclease superfamily to which IS*91* and ISCRs (Insertion sequence common regions [40]) belong as well. IS*91* and ISCRs are postulated to transpose their ssDNA sequence with a rolling circle replication mechanism that starts at a specific site named *Ori*-IS and ends at a termination sequence *ter*-IS. However, high frequency of termination failure at the *ter*-IS site can be observed, leading to a one ended-transposition, mobilizing adjacent sequences at the 3′ end of the IS element [37]. Although this mechanism could explain the associated antibiotic genes commonly found downstream ISCRs, it cannot explain those lying at its upstream part [41]. Perhaps, a common minor transposition mechanism for ISCRs and IS*200/605*, other than the one already established for IS*200/605* transposition, exists allowing mobilization of adjacent genes to the IS elements in both directions. If this is true, this may allow the transfer of a CALIN without requiring the activity of an integron integrase for excising and integrating separate gene cassettes. Definitely, this needs a lot of investigation and experimental work to be verified.

It is interesting to note the clustering of different genetic elements (*att*C sites, group II introns and IS*200/605*) that require single stranded and secondary structures for function. These elements have been linked to replication as one of the main sources for ssDNA [1, 28, 32, 42]. Further experimental studies should be performed to delineate the interaction between the gene cassettes, group II introns and IS*200/605* elements from hypersaline environments.

### Abundance of toxin-antitoxin (TA) systems in hypersaline integron-associated structures

Finally, our analysis showed abundance of TA systems belonging to different classes in all identified arrays. The abundance of TA systems as gene cassettes within integrons has already been observed in different studies [14]. It is hypothesized that TA systems could have a role in maintaining the integrity of these integrons by preventing deletions of existing arrays [14, 43]. Nonetheless, the accumulation of 6 different TA systems within the identified *H. halochloris* CALIN is intriguing. In fact, both H.ha.F1 and H.ha.F2 truncated introns were inserted into gene cassettes composed of a TA operon, although in case of H.ha.F2, a frameshift due to a one nucleotide deletion in the HicA family toxin ORF is observed. In addition, 3 TA systems of the BrnT/A family were detected within the CALIN. The claimed hypothesis that TA systems are important for the integrity and maintenance of the adjacent chromosomal structures indicates that adjacent gene cassettes and even secondary structures have unraveled essential roles. Moreover, the large number of expressed TA systems in a genome was found to have a role in increasing the population of persisters that can survive under different stress conditions [43]. It is therefore not surprising that the detected TA systems in the metagenome and genome from hypersaline environments would support the adaptation and growth of the persistent halophiles. ParE toxins of TA systems, which were identified in TSL1, TSL2 and *H. halochloris* CALIN, were shown to induce DNA damage, which in turn induces an SOS response, activating DNA repair mechanisms where ssDNA stretches are formed allowing different transpositions and recombination events to take place [43]. Similarly the identified TA cassettes from hypersaline environments can increase mobilization of different MGEs such as integron gene cassettes, prophages and transposons [43].

## Conclusions

Integrons and integron-like sequences have been particularly associated with Group IIC-*att*C introns. In this study we identified a Group IIC-*att*C from the hypersaline Tanatar-5 Soda lake metagenome in Russia. We have also detected different classes of group IIB introns within gene cassette arrays in the same metagenome and in a CALIN in the extreme halophile *H. halochloris*. These findings could help decipher the role of group II introns associated with integrons or integron-associated sequences in hypersaline environments. A new insertion sequence IS*Hahl1*, belonging to IS*200/605* elements was also identified adjacent to *H. halochloris* CALIN. The clustering of different MGEs, particularly those requiring single-stranded secondary structures for their function, suggests interplay between these different elements and cellular processes such as replication, transcription and horizontal gene transfer of prokaryotes residing in hypersaline environments. The abundance of toxin-antitoxin systems in all our studied gene cassette arrays, either as gene cassettes or right after the last *att*C site, strengthens their potential role in maintaining the integrity of the adjacent arrays, enhancing the mobility of adjacent mobile elements and increasing the persistence of the cells to adapt to their hypersaline and alkaline environments.

## Methods

### Analyzed samples

We analyzed publicly available metagenomic assemblies from different hypersaline environments (28 assemblies of a total of 1,236,831,758 bp and 658,054 contigs) in addition to completely or partially sequenced genomes of halophilic bacteria (24 complete and 33 partial with a total size of 202.81 Mb) and archaea (25 complete and 22 partial with a total size of 166.02 Mb). Table 1 shows all analyzed assemblies, whereas a list of halophilic bacteria and archaea was obtained from the Halodom database [44] in November 2019: “halodom.bio.auth.gr” (Additional file 2 Table S2 and S3). The analyzed metagenomic assemblies were all available already assembled hypersaline metagenomes on NCBI or from our lab. For comparative reasons, metagenomic assemblies from 22 marine and 7 freshwater environments (1,750,281,271 bp and 1,444,498 contigs) were subjected to the same analysis (Additional file 2 Table S4). The marine assembled metagenomes were selected from different geographical locations, different depths if applicable with a tendency towards choosing those with smaller number of contigs for easier processing. In case of freshwater assemblies, we used all publicly available assembled metagenomes on NCBI.

**Table 1:** Analyzed metagenomic assemblies from different hypersaline environments

Site	Description	Assembly Accession number or reference	Total assembled sequence length	Number of contigs
GR	Grendel Spring, Yellowstone National Park, Wyoming, USA	GCA_900244995.1	33,631,634	11,151
GNM1	Guerrero Negro mat, Mexico 0-1 mm depth	GCA_000206585.1, [45, 46]	8,530,607	11,351
GNM2	Guerrero Negro mat, Mexico 1-2 mm depth	GCA_000206565.1, [45, 46]	7,390,978	10,551
GNM3	Guerrero Negro mat, Mexico 2-3 mm depth	GCA_000206545.1, [45, 46]	8,209,846	11,423
GNMt4	Guerrero Negro mat, Mexico 3-4 mm depth	GCA_000206525.1, [45, 46]	8,130,049	11,724
GNM5	Guerrero Negro mat, Mexico 4-5 mm depth	GCA_000206505.1, [45, 46]	9,689,398	14,128
GNM6	Guerrero Negro mat, Mexico 5-6 mm depth	GCA_000206485.1, [45, 46]	8,291,075	11,380
GNM7	Guerrero Negro mat, Mexico 6-10 mm depth	GCA_000206465.1, [45, 46]	9,759,240	13,649
GNM8	Guerrero Negro mat, Mexico 10-22 mm depth	GCA_000206445.1, [45, 46]	7,914,434	11,356
GNM9	Guerrero Negro mat, Mexico 22-34 mm depth	GCA_000206425.1, [45, 46]	8,308,787	11,596
GNM10	Guerrero Negro mat, Mexico 34-49 mm depth	GCA_000206405.1, [45, 46]	7,132,956	10,297
ATII SDM	Atlantis II Deep Brine Sediment, Red Sea	[47–49]	40,413,330	41,726
DD SDM	Discovery Deep Brine Sediment, Red Sea	[47–49]	52,421,642	51,829
Th	Thetis Mediterranean deep-sea hypersaline lakes	GCA_001684355.1	13,102,297	10,347
ATII INF	Atlantis II Deep Brine interface, Red Sea	[49, 50]	16,014,945	24,317
DD INF	Discovery Deep Brine interface, Red Sea	[49, 50]	11,647,401	18,413
KD UINF	Kebrit Deep Upper interface, Red Sea	[49, 50]	42,652,688	45,750
KD LINF	Kebrit Deep Lower interface, Red Sea	[49, 50]	50,280,352	74,666
ATII LCL	Atlantis II Deep Brine, Lower convective layer, Red Sea	[49, 50]	46,518,597	43,555
ATII UCL	Atlantis II Deep Brine,Upper convective layer, Red Sea	[49, 50]	21,343,827	29,592
DD BR	Discovery Deep Brine, Red Sea	[49, 50]	12,244,355	18,850
KD BR	Kebrit Deep Brine, Red Sea	[49, 50]	35,162,057	74,666
TSL	brine of Lake Tanatar-5 (Soda Lake), Russia: Kulunda steppe	GCA_001564335.1	193,970,398	19,350
TTCSL	brine of Tanatar trona crystallizer (Soda Lake), Russia: Kulunda steppe	GCA_001563815.1	106,596,264	9426
PSL	brine of Picturesque Lake (Soda Lake), Russia: Kulunda steppe	GCA_001564315.1	251,189,393	25,098
Ty	Lake Tyrrell, Victoria, Australia	GCA_000347535.1, [51, 52]	62,549,170	15,008
Na	Namib Desert Hosabes playa, Namibia	GCA_001543535.1	10,867,082	11,304
BSL	brine of Lake Bitter-1 (Soda Lake), Russia: Kulunda steppe	GCA_001563825.1	152,868,956	15,551

### Identification of integrons and CALINs

Integron finder version 2.0 [18] was used to search for complete integrons, Integron integrase genes (*int*I) and CALINs in hypersaline metagenomic assemblies and genomes of different halophiles. We used the option “local detection” on the command line with all contigs and an 8 kb distance threshold between successive identified *att*C sites to ensure the detection of all potential *att*C sites. At least 2 *att*C sites should be detected within the 8 kb threshold to be reported as a positive result. A search for integron cassette promoters (P_C_) and primary recombination sites (*att*I) for known integron classes (1, 2 and 3) was also performed.

### Identification of group II introns

Identified sequences were further inspected by running BLAST search of all identified ORFs within gene cassettes against NCBI nr BLAST database. ORFs identified as group II RT/maturase were further analyzed by blastx against group II intron database (http://webapps2.ucalgary.ca/~groupii/) [25, 26] and their amino acid sequences were aligned with close hits in order to identify IEP different domains that were defined in group II intron database (http://webapps2.ucalgary.ca/~groupii/html/static/orfalignment.php) [25, 26]. Identification of intron boundaries was done by the MFOLD webserver, which folds the introns RNA structure [36] based on the known secondary structures of group II intron classes, that showed the high similarity to our newly identified introns. First, for each identified Group II intron RT, the region downstream of the ORF was aligned with 3–6 sequences from close hits obtained by blast using MUSCLE [53, 54]. This was done to identify the most conserved DV in addition to DVI and the 3′ boundary of the intron. This was followed by searching for the basal stem of DIV by looking for a sequence complementary to the sequence just upstream DV within the ORF or within 200 bp upstream of the ORF start codon. Identification of the 5′ domains (DI, DII and DIII) was mainly done by searching for a putative 5′ boundary following the consensus sequence GUGYG and folding into a structure similar to the consensus structure of the identified group II intron class. Even with the low sequence conservation in upstream domains, multiple sequence alignment with close introns helped in determination of the final folding structure. Moreover, exon binding sequences (EBS1, 2 and 3) and sequences involved in tertiary structures such as α-α’, β-β’, δ-δ’, Ɛ-Ɛ’ and γ-γ’ Watson-Crick base pairs, ζ-ζ’ and η-η’ tetraloop-receptor interactions and ĸ-ĸ’ and λ-λ’ non Watson-Crick interactions [6] were determined manually whenever applicable. The final secondary structure was then depicted using Pseudoviewer3 [55].

Sequence logos of intron boundaries and 5′ and 3′ exons of each identified intron with its closest homologues (obtained by Blastx against group II intron database) were illustrated using WebLogo ver. 2.8.2 [56].

Detection of introns upstream hairpin structures was done using MFOLD [36], respectively.

### Insertion sequences identification

ISfinder [23] was used to search for insertion sequences within contigs or genomes at which integrons or CALINS were identified. ISEScan pipeline [29] was also used for further inspection of insertion sequences within *H. halochloris* DSM 1059 genome.

### ORFs annotation and promoter predictions

All predicted ORFs within identified gene cassettes were manually curated and annotated based on Blastx results against NCBI nr database. Search for promoters for TA systems genes, IEP ORFs and within group II introns was done using bprom tool [57].

### Phylogenetic analysis

34 bacterial IEPs from different classes were aligned to the 4 identified IEPs in this study using MUSCLE [54], along with Mitochondrial IEP from Liverwart *Marchantia polymorpha* which was used as an outgroup. Molecular phylogenetic analysis was done with MEGA7 [58] using the Maximum Likelihood with WAG substitution model. The tree was drawn to scale, with branch lengths depicting the number of substitutions per site. Statistical support of the tree was done by bootstrap analyses with 1000 samplings.

### Determination of *H. halochloris* leading and lagging strands

GammaBOriS tool specifically designed for identification of origin of replication (*Ori*C) sequences in gammaproteobacterial chromosomes [30] was used for identification of probable *H. halochloris Ori*C. Based on the approach used by Mao et al. [59]. The position of the replication termination site was roughly calculated as half of the genome DNA sequence starting from the identified *Ori*C. The leading and lagging strands of each half was then determined based on the knowledge that the leading strands encodes for a much larger number of genes than the lagging strand [59].

## Supplementary Information

**Additional file 1 Figure S1.** A: Multiple sequence alignment of UHB.F1 with closely related IEP showing RT domains (RT0–7) and X domain and the highly conserved YADD motif within RT5 domain. B. Schematic representation of UHB.F1 IEP showing relative positions of its RT domains (0–7) and X domain. **Figure S2.** Amino acid sequences of identified IEPs showing internal stop codons as “*” and frameshifts as “/”. Positions within contigs or genome are indicated as well. **Figure S3.** A: Multiple sequence alignment of UHB.I2 with closely related IEP showing RT domains (RT0–7) and X domain and the highly conserved YADD motif within RT5 domain. B. Schematic representation of UHB.I2 IEP Schematic representation of UHB.F1 IEP showing relative positions of its RT domains (0–7) and X domain. **Figure S4.** Multiple sequence alignment of H.ha.F1 and H.ha.F2 IEP with closely related IEP from bacterial class E showing missing RT1, 2,3 and part of RT4 in both ORFs and missed RT0 in H.ha.F2 as well. A internal stop codon in H.ha.F1 is shown as an asterisk. The highly conserved YADD motif is within RT5 domain. **Figure S5.** Identified introns’ DNA sequences with their positions within their contigs (TSL1 and TSL2) or genome (*H. halochloris*). Domains are shown in different colors: DI, DII, DIII, DIV, DV, DVI, ORF underlined. Putative promoters are either underlined with a zigzagged line (same orientation) or with a dotted line (opposite orientation). Intron boundaries are colored in cyan. **Figure S6.** Folding of DV and DVI RNA of truncated UHB.F1 within TSL1 metagenomic contig. **Figure S7.** 5′ exon secondary structure of UHB.I2. *att*C top strand (ts) secondary structure upstream of UHB.I2. **Figure S8.** Folding of DV and DVI RNA of fragmented group II introns identified within a CALIN in *H. halochloris.* **Figure S9.** Left and right end hairpin structures of IS*Hahl1* compared to IS*CARN6*, both belonging to IS*605* group of IS*200/605* superfamily. A conservation in secondary structure and to a lesser extent in primary structure is shown between left and right ends of both IS elements. **Figure S10.** Secondary structure of putative *att*C sites top strands (ts) and bottom strands (bs) undetected by integron Finder upstream H.ha.F1 and H.ha.F2. A: Atypical *att*C upstream H.ha. F1, B: Putative *att*C upstream H.ha.F2.

**Additional file 2 Table S1**. Genetic elements description and position within gene cassette arrays in examined sites. **Table S2.** Analyzed complete and partial bacterial halophilic genomes. **Table S3.** Analyzed complete and partial archaeal halophilic genomes. **Table S4.** Analyzed metagenomic assemblies from different marine, freshwater and hydrothermal vents environments.

## Data Availability

Accession numbers to all used publically available assembled metagenomes are included in methods section (Table 1) and Additional file 2 Table S4. Assembled metagenomes from Red Sea brine pools and from hydrothermal vents are available from the corresponding author on reasonable request. List of halophilic bacteria and archaea was obtained from the Halodom database [44] in November 2019: (halodom.bio.auth.gr) and accession numbers of analyzed genomes are included in Additional file 2 Tables S2 and S3. Group II introns sequences and their corresponding IEPs were obtained from group II intron database (http://webapps2.ucalgary.ca/~groupii/html/static/intro.php) [25, 26]. Identified IS during this study is submitted at ISfinder (https://isfinder.biotoul.fr/) [23] under the name IS*Hahl*1. All data generated during this study are included in this published article and its additional files.

## Abbreviations

bs: Bottom strand
CALIN: Clusters of *att*C sites lacking a neighboring integron-integrase
cDNA: Complementary DNA
CL: Chloroplast-like
dsDNA: Double-stranded DNA
EBS: Exon binding site
En: Endonuclease
IBS: Intron binding site
IEP: Intron encoded Protein
intI: Integron integrase
IS: Insertion Sequence
MGE: Mobile genetic element
ML: Mitochondrial-like
ORF: Open reading frame
qRT-PCR: Quantitative real time reverse transcriptase ploymerase chain reaction
RNA-seq: RNA sequencing
RNP: Ribonucleoprotein
RT: Reverse transcriptase
ssDNA: Single-stranded DNA
TA: Toxin-Antitoxin
