# Supplementary material for: The association of group IIB intron with integrons in hypersaline environments
Source: Mob DNA. 2021 Mar 1;12:8. doi: 10.1186/s13100-021-00234-2 (PMC7923331; doi:10.1186/s13100-021-00234-2)
Supplement: Supplementary file 1 — Additional file 1 Figure S1. A: Multiple sequence alignment of UHB.F1 with closely related IEP showing RT domains (RT0–7) and X domain and the highly conserved YADD motif within RT5 domain. B. Schematic representation of UHB.F1 IEP showing relative positions of its RT domains (0–7) and X domain. Figure S2. Amino acid sequences of identified IEPs showing internal stop codons as “*” and frameshifts as “/”. Positions within contigs or genome are indicated as well. Figure S3. A: Multiple sequence alignment of UHB.I2 with closely related IEP showing RT domains (RT0–7) and X domain and the highly conserved YADD motif within RT5 domain. B. Schematic representation of UHB.I2 IEP Schematic representation of UHB.F1 IEP showing relative positions of its RT domains (0–7) and X domain. Figure S4. Multiple sequence alignment of H.ha.F1 and H.ha.F2 IEP with closely related IEP from bacterial class E showing missing RT1, 2,3 and part of RT4 in both ORFs and missed RT0 in H.ha.F2 as well. A internal stop codon in H.ha.F1 is shown as an asterisk. The highly conserved YADD motif is within RT5 domain. Figure S5. Identified introns’ DNA sequences with their positions within their contigs (TSL1 and TSL2) or genome (H. halochloris). Domains are shown in different colors: DI, DII, DIII, DIV, DV, DVI, ORF underlined. Putative promoters are either underlined with a zigzagged line (same orientation) or with a dotted line (opposite orientation). Intron boundaries are colored in cyan. Figure S6. Folding of DV and DVI RNA of truncated UHB.F1 within TSL1 metagenomic contig. Figure S7. 5′ exon secondary structure of UHB.I2. attC top strand (ts) secondary structure upstream of UHB.I2. Figure S8. Folding of DV and DVI RNA of fragmented group II introns identified within a CALIN in H. halochloris. Figure S9. Left and right end hairpin structures of ISHahl1 compared to ISCARN6, both belonging to IS605 group of IS200/605 superfamily. A conservation in secondary structure and to a lesser extent in prima [file 13100_2021_234_MOESM1_ESM.docx]

Fig. S1. A: Multiple sequence alignment of UHB.F1 with closely related IEP showing RT domains (RT0-7) and X domain and the highly conserved YADD motif within RT5 domain. B. Schematic representation of UHB.I1 IEP showing relative positions of its RT domains (0-7) and X domain.


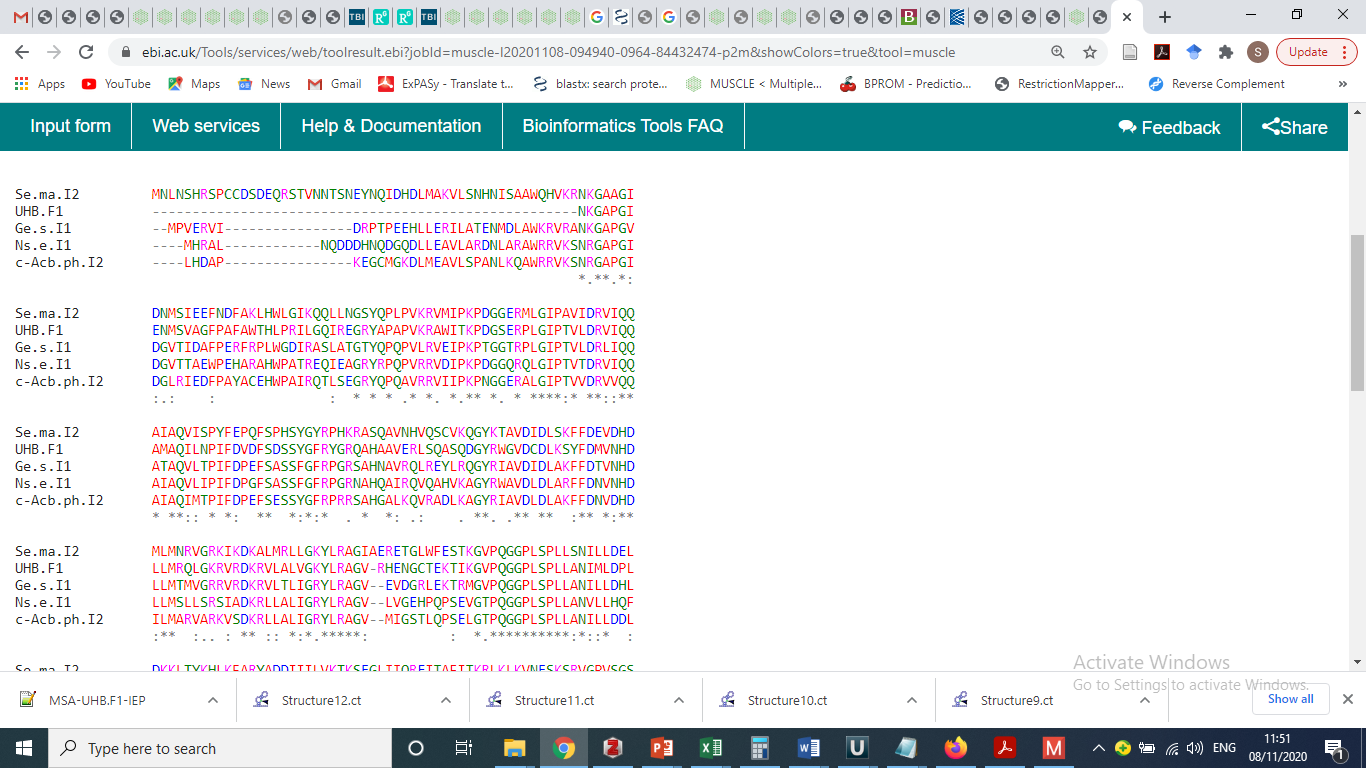

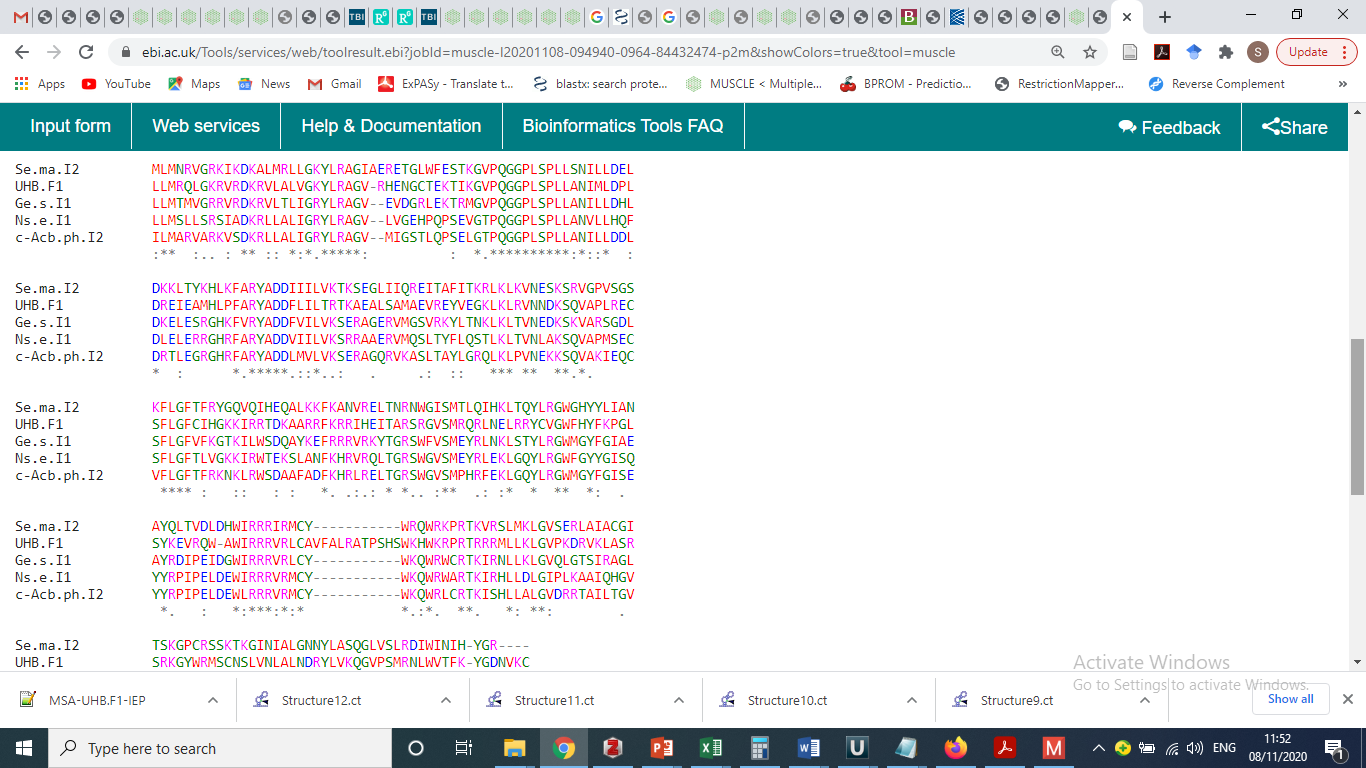

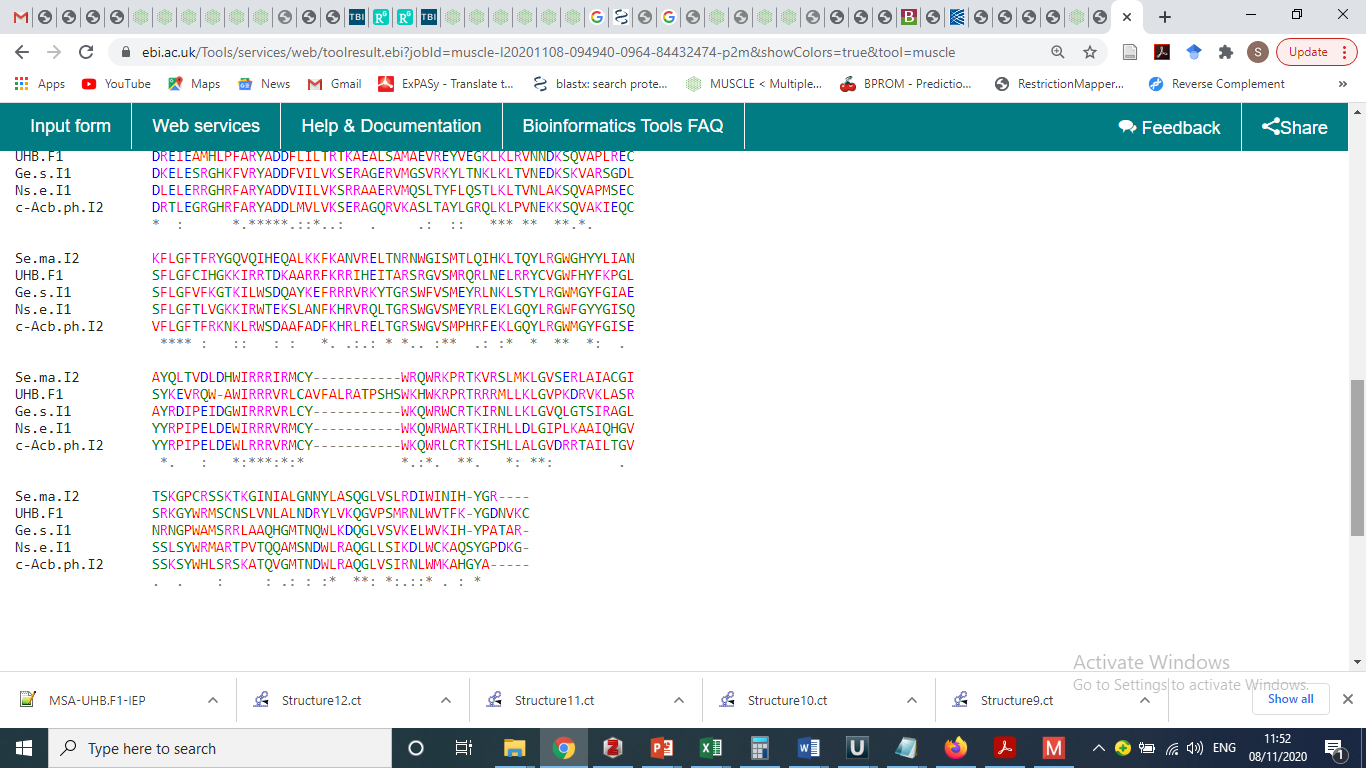


**0**

**1**

**2a**

**3**

**4**

**5**

**6**

**X**

**2**

**7**

A

0 1 2 2a 3 4 5 6 7 X

B

Fig. S2. Amino acid sequences of identified IEPs showing stop codons as “*”, frameshifts as “/” and insertions underlined. Positions within contigs or genome are indicated as well.

>IEP- UHB.F1-3872..5111

NKGAPGIENMSVAGFPAFAWTHLPRILGQIREGRYAPAPVKRAWITKPDGSERPLGIPTVLDRVIQQAMAQILNPIFDVDFSDSSYGFRYGRQAHAAVERLSQASQDGYRWGVDCDLKSYFDMVNHDLLMRQLGKRVRDKRVLALVGKYLRAGVRHENGCTEKTIKGVPQGGPLSPLLANIMLDPLDREIEAMHLPFARYADDFLILTRTKAEALSAMAEVREYVEGKLKLRVNNDKSQVAPLRECSFLGFCIHGKKIRRTDKAARRFKRRIHEITARSRGVSMRQRLNELRRYCVGWFHYFKPGLSYKEVRQWA/WIRRRVRLC/AVFALRATPSHSWKHWKRPRTRRRMLLKLGVPKDRVKLASRSRKGYWRMSCNSLVNLALNDRYLVKQGVPSMRNLWVTFKYGDNVKC*

>IEP-UHB.I2-c(5223..6725)

MIPDKGSALRNMPRNWRSLDWDAAERHVKRLQVRIAKAVEEKKWGKVKALQWTLTHSFYAKALAVRRVTRNKGARTPGIDKARWRTDGRKLAAVLQLKRHGYRAKALRRIYILKKNGKKRPLSIPTMNDRAMQALYALALIPVAEALADPNSYGFREGRCCQDALEQCFVILARRVSPGWILEADIKGCFDNISHEWLMNHIPLDKSILRQWLEVGYIEEGEWFRSEAGTPQGGIVSPILANLTLNGLEKAIKASVPSTETGVNVVRYADDFIVTARSPERLTETIRPVIERFLAERGLSLSEEKTKITSIDEGFDFLGQNARKYEGKLLIKPSKTSTQGLLDKVRLIIDAHKGKSAERLIKVLNPVIRGWANYHRHSVCAQTFYYIDYVISGALFRWIRKRNQNKSKSWIVWKHFRSPLDKSGTFCAKSKNKKGQTVYYHLQKALNIPRALHRKVIGKAHPYQPEKAEYFAKRQLKRYRTKGRMSQPMQWIQAHLGFQP*

> IEP-H.ha.F1-1187659.. 1188685

VPEGNTKHPQWRGCGGLAGSSGRGMQGEIRRRTREAPKGSCGGEGRQGPTAIETRRGNLETKRYRTRRVRRCYIPKEDGGERPLGIPAVEDRLLQAACARILTAIYEADFLDGSYGYRPGKSAKDAVADLGST/LHYALDLWFEQVVKPRCRGQALLVRYADDYVCAFQFQEDAQRFYRAVPRRLGRFGLQVAPEKTRLMRFSRFHPGLRRRFGFLGFELNWSRDRRGELRVMKRTARKKLQAAKRRLKGWIRANRHLPGRVFIQELNRRLVGHYNYFGLRSNEQGLGSYHIFAIRCAFK*LNRRGGKRSSFNWAQYIEALRKLGVAQPRITERQRAHGVFA*

> IEP-H.ha.F2 -1196335..1196964

YLHYALDLWFERVVKPRCRGQALLVRYADDYVCAFQFQEDAQRFYRAVPRRLGRFGLQVAPEKTRLMRFSRFHPGLRRRFGFLGFELYWSRDRRGELRVMKRTVRKKLQAAKRRLKGWIRANRHLPGRVFIQELNRRLVGHYNYFGLRSNEQGLESYYIFATRCAFKWLNRRGGKRSSFNWAQYIAALRKLGVEQPRITERQRAHAVFA*

Fig. S3. A: Multiple sequence alignment of UHB.I2 with closely related IEP showing RT domains (RT0-7) and X domain and the highly conserved YADD motif within RT5 domain. B. Schematic representation of UHB.I2 IEP Schematic representation of UHB.I1 IEP showing relative positions of its RT domains (0-7) and X domain


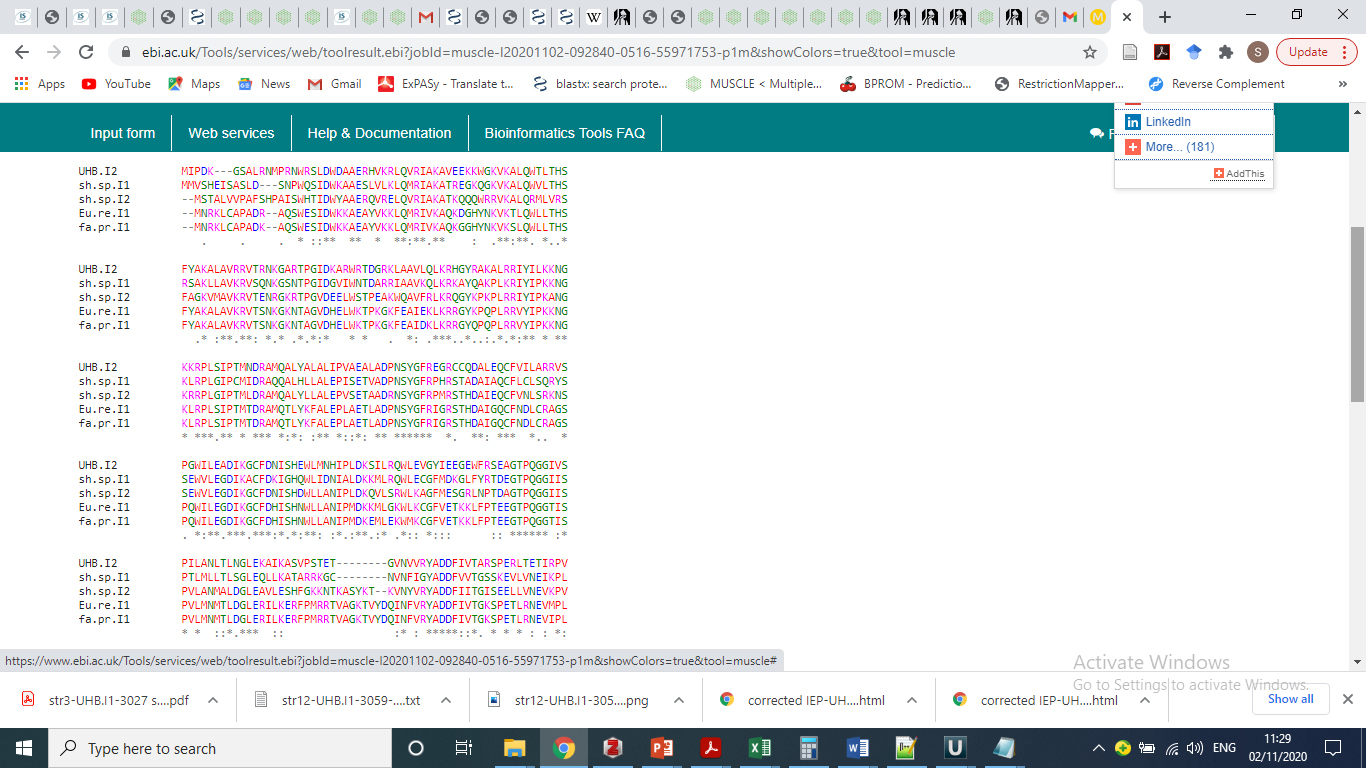

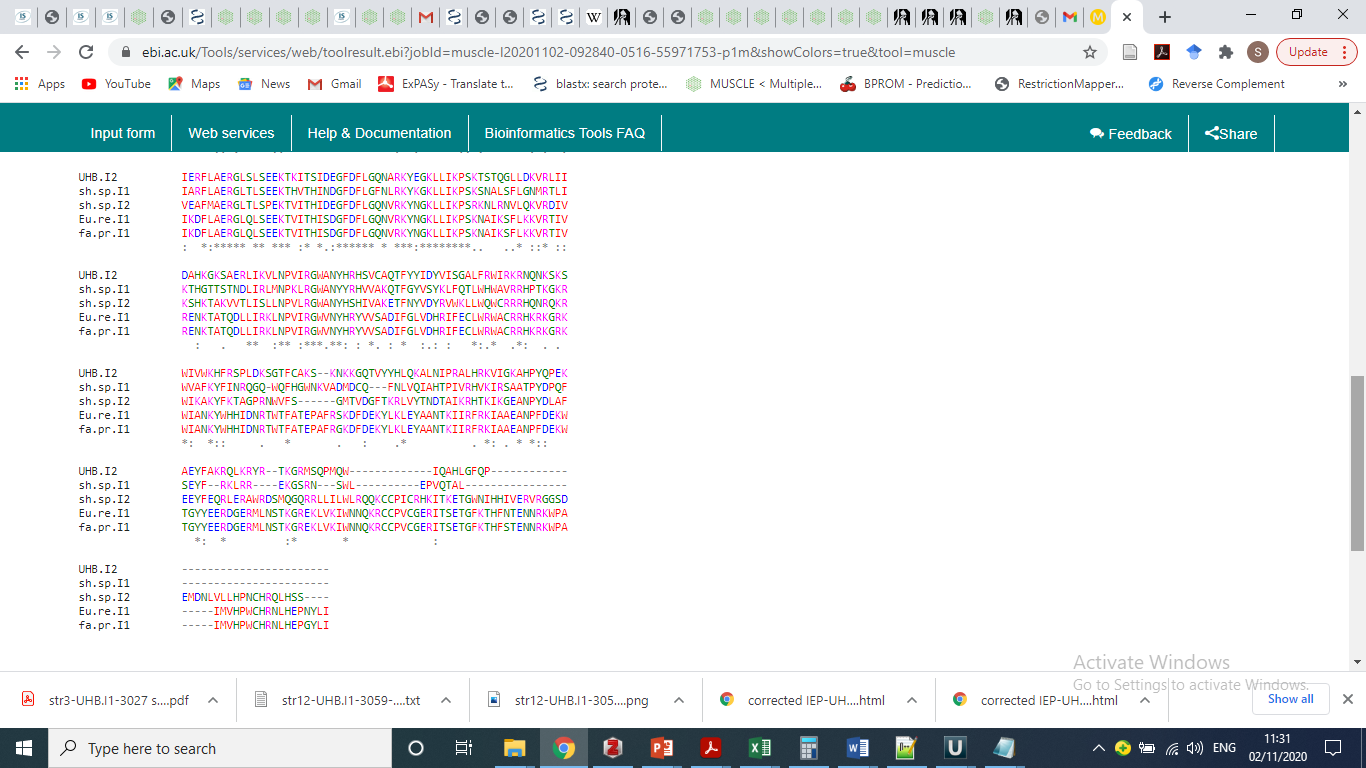


**0**

**1**

**2a**

**3**

**4**

**5**

**6**

**7**

**2**

**X**

**3**

**7**

**6**

**X**

A

0 1 2 2a 2/3 3 4 5 5/6 6 7 X

B

Fig. S4. Multiple sequence alignment of H.ha.F1 and H.ha.F2 IEP with closely related IEP from bacterial class E showing missing RT1, 2,3 and part of RT4 in both ORFs and missed RT0 in H.ha.F2 as well. A internal stop codon in H.ha.F1 is shown as an asterisk. The highly conserved YADD motif is within RT5 domain.


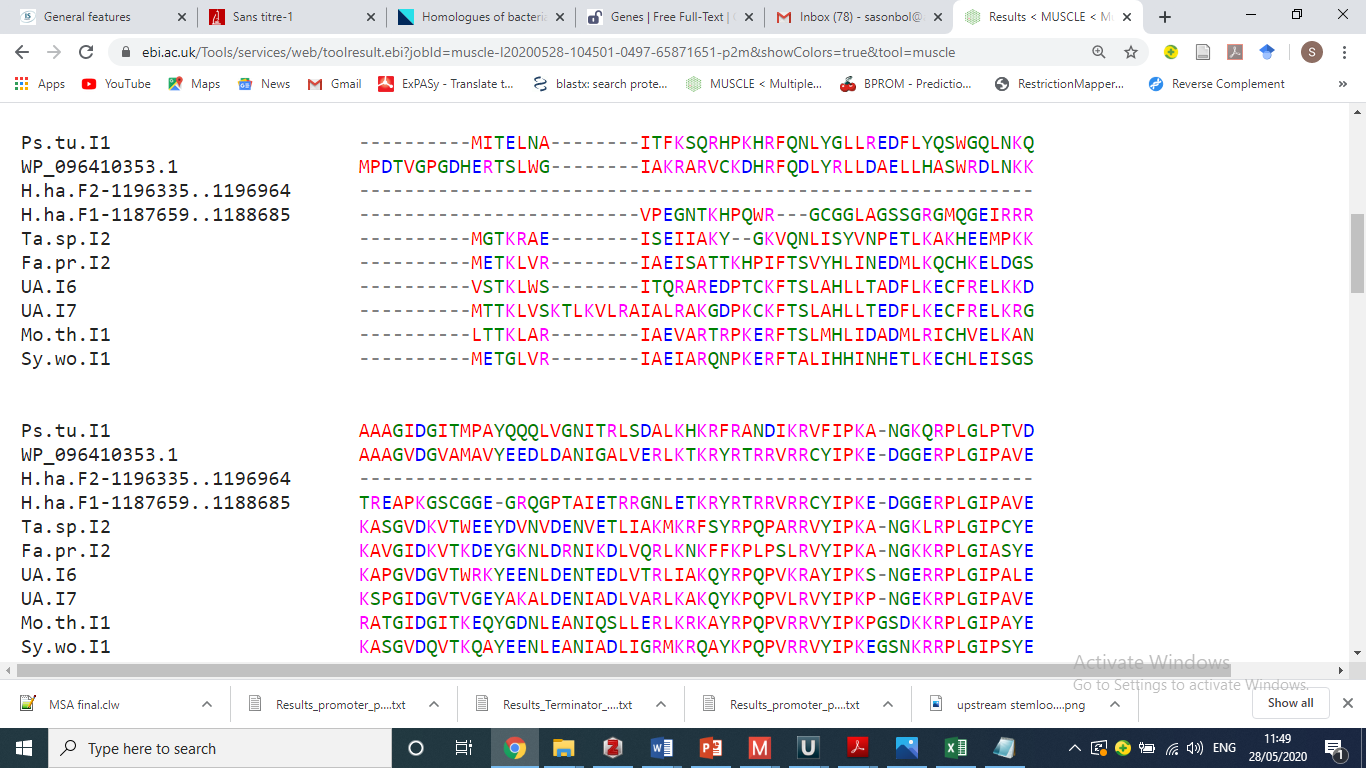

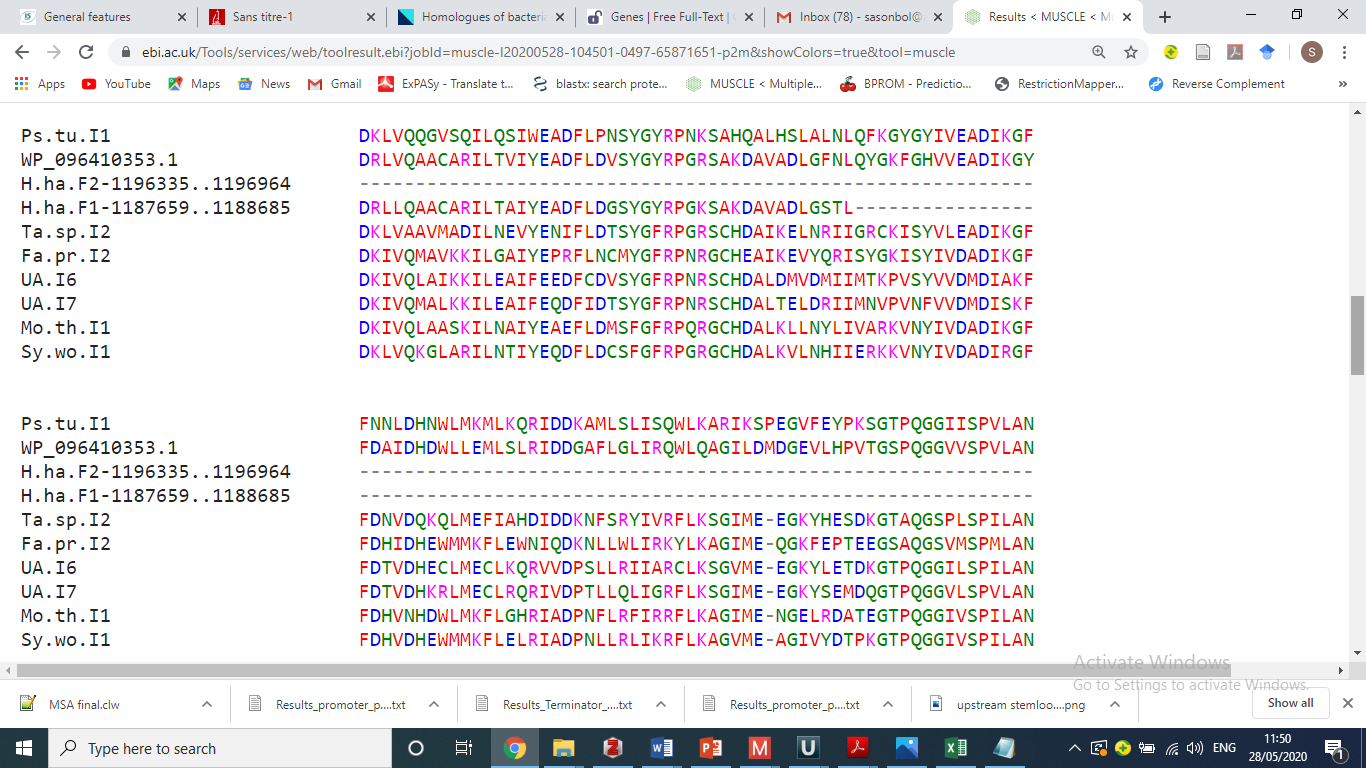


**0**

**1**

**2a**

**3**

**4**

**2**


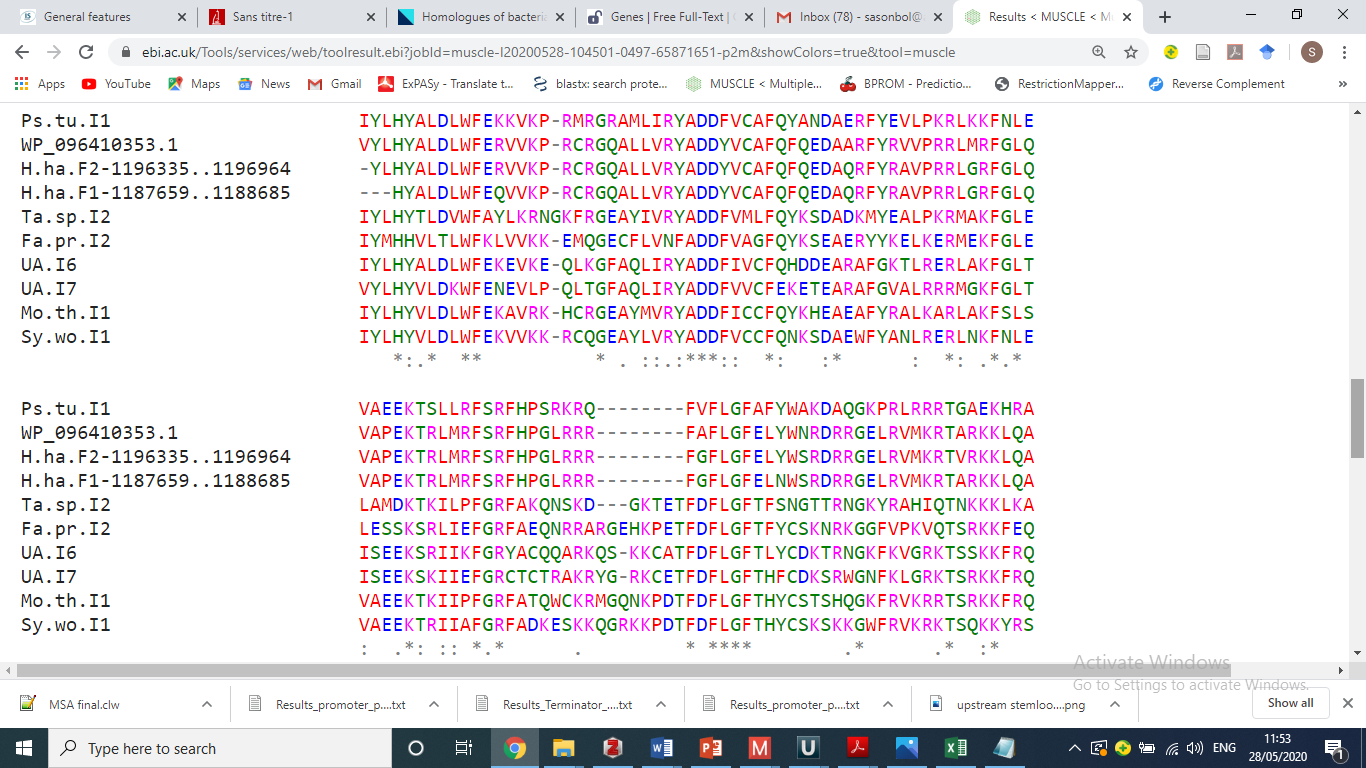

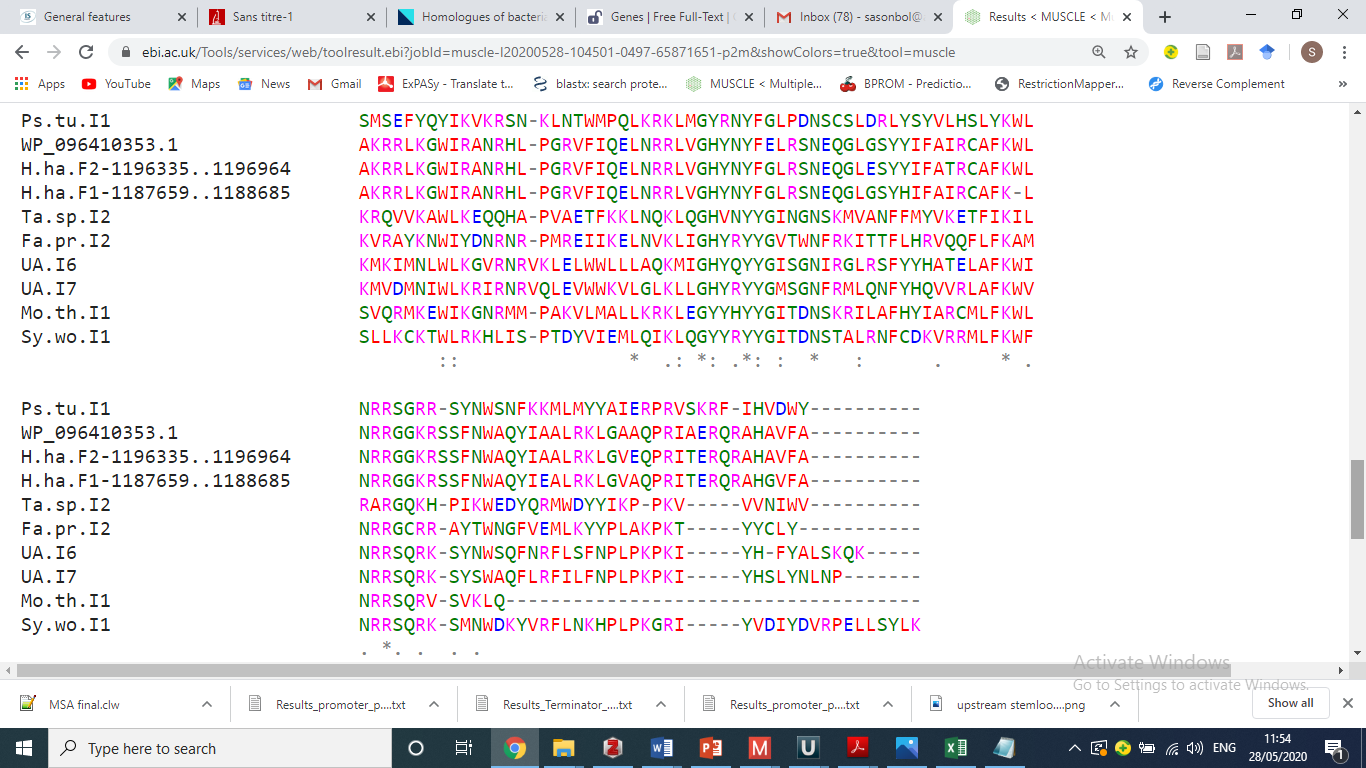


**5**

**6**

**7**

**X**

*

Fig. S5. Identified introns’ DNA sequences with their positions within their contigs (TSL1 and TSL2) or genome (*H. halochloris*). Domains are shown in different colors: DI, DII, DIII, DIV, DV, DVI, ORF underlined. Putative promoters are either underlined with a zigzagged line (same orientation) or with a dotted line (opposite orientation). Intron boundaries are colored in cyan.

UHB.F1-fragmented_intron-3872..5204

AACAAAGGAGCACCGGGGATCGAGAACATGTCGGTCGCCGGGTTTCCGGCGTTTGCATGGACGCACCTGCCAAGAATATTGGGGCAGATTCGGGAGGGGCGCTATGCCCCTGCTCCGGTTAAAAGAGCTTGGATCACCAAACCGGACGGAAGCGAACGCCCCCTGGGCATACCGACCGTTCTGGACCGGGTGATCCAGCAAGCCATGGCTCAAATCCTCAATCCCATCTTTGATGTGGATTTCAGCGACAGCAGCTATGGTTTCAGATACGGACGCCAGGCTCACGCCGCCGTCGAGCGGTTGAGTCAGGCGAGTCAGGACGGTTACCGTTGGGGAGTGGACTGCGACCTGAAGTCCTACTTCGACATGGTAAACCACGACCTGTTGATGCGTCAGCTGGGTAAGCGAGTCCGGGACAAACGCGTTCTCGCCCTGGTCGGCAAATACCTGCGTGCAGGTGTCAGGCATGAGAACGGTTGCACGGAGAAAACGATCAAAGGCGTTCCTCAGGGAGGCCCGTTGTCTCCGCTGCTTGCCAACATCATGCTCGACCCGCTCGACCGGGAAATCGAGGCGATGCACCTGCCGTTTGCCCGCTACGCGGATGATTTCCTCATCCTCACCCGCACCAAAGCCGAGGCGCTGAGCGCCATGGCCGAAGTCCGGGAGTATGTGGAGGGAAAACTGAAGTTGCGGGTTAATAACGACAAAAGTCAGGTTGCTCCCTTGAGGGAATGCAGCTTCCTTGGGTTCTGTATCCACGGGAAGAAAATCCGGCGGACCGATAAGGCAGCCCGGAGATTCAAACGCCGGATACATGAGATTACCGCCCGCAGTCGGGGCGTCTCGATGAGGCAACGCCTCAACGAACTCCGGCGTTATTGCGTGGGGTGGTTTCATTACTTCAAGCCGGGCCTTTCCTATAAGGAAGTCCGACAGTGGGCCTGTGGATACGCAGGCGCGTGCGCCTGTGCTAGCCGTCTTCGCCCTGCGGGCTACGCCGAGCCACAGCTGGAAACACTGGAAGCGGCCGCGAACGCGGAGACGAATGCTCCTGAAACTCGGCGTCCCTAAAGACCGGGTGAAGCTGGCATCCCGCTCCCGCAAGGGCTATTGGCGAATGTCGTGCAACAGTCTGGTCAACCTGGCCCTCAATGATCGTTATCTGGTAAAACAAGGGGTACCGTCGATGCGGAACCTCTGGGTGACCTTCAAATATGGAGATAACGTCAAGTGCTAGTCTCCGGTCACTGATTCTTCGGAACCGCCGTGATACGGACCCGTATGTCCGGTGGTGTGGGGGCCGGGGAGTTAACGCTCCCGGCTACCCGAT

UHB.I2- intron-c(5096..7296)

TTGCGACATGATGTTACGCAAGATACTGATTAACAACAACTATGATATAATGGAAAACTGATTAATCCTCGCATTCCACTGCGAGTTCGCCAAGGGGCGTGCGCCCTGTCGGGCGGTTTCGGTTCGCCGGAGCTACTGGCAGGGGGTGCGAAGCCTTCCTGGAATGTAATGATCCCTGGAGCCTCTGCCGGGGGGATTGCTAGCGACGGGCGGTATGGTGAGATTAGACGAAAGGTCGAAAGCGCCGTAAAGGAAACGAGCACAAAAGAGGCTAATGAGCTCAGCCAAAAGGCAAGTAGTCAGGTTTCGGGTGTAGAACGTGCCCGGACGTGGATGTTGAACTGCCGGCGTAACAGACCTCACCTAACCCGCTCTCGTATCTTGTGTGGAACATGGGAACCCGGATCTTCTTCCCTCCGCGGGAAGGCGTCACCGCAAGGCGCGTATCGGAGATCTGGATTGAGAGGTTCGAAAAAGCCAATGCCCGCCTGCAATGGGTGCGGATATGCCCACGTCGAACTGGTGTTTCTTTGTGAGAGAACCTGATAAACCGATGAATCGCAAAAAGCAGATGATCCCCGACAAAGGGAGTGCATTGCGAAACATGCCACGAAACTGGCGCTCCCTTGACTGGGACGCCGCCGAACGGCACGTTAAACGGCTCCAGGTGCGTATCGCAAAGGCAGTTGAAGAAAAGAAATGGGGCAAGGTGAAAGCCTTGCAATGGACGCTGACCCACTCCTTTTACGCCAAAGCTTTGGCCGTAAGGAGAGTCACGCGCAACAAGGGAGCTCGCACGCCCGGCATCGACAAAGCCCGCTGGAGAACCGACGGACGAAAACTCGCTGCCGTGCTCCAGCTCAAACGCCACGGCTACCGAGCCAAGGCGTTGCGTAGAATCTATATCCTAAAGAAGAATGGCAAGAAACGTCCCCTGAGTATCCCGACAATGAACGACCGGGCAATGCAGGCGCTTTACGCGCTTGCGCTGATACCGGTAGCCGAAGCACTGGCCGACCCGAACTCCTACGGATTTCGCGAAGGACGCTGCTGTCAGGACGCTCTCGAACAATGCTTCGTCATCCTGGCCAGACGGGTCTCCCCCGGATGGATACTGGAGGCGGACATCAAAGGCTGTTTCGACAACATCAGCCACGAATGGCTGATGAACCATATTCCGCTGGACAAAAGCATTCTGCGTCAATGGCTGGAAGTTGGTTACATAGAGGAAGGAGAATGGTTCCGGTCGGAAGCGGGAACTCCGCAAGGCGGAATCGTCTCGCCAATCCTCGCCAATCTCACACTCAACGGACTCGAAAAAGCCATCAAGGCATCGGTCCCGAGCACAGAGACTGGTGTTAACGTAGTTCGGTATGCCGACGACTTCATTGTCACGGCAAGGTCGCCGGAAAGACTGACGGAGACGATTCGACCCGTAATCGAGCGATTTCTCGCCGAACGCGGGTTGAGTCTTTCCGAGGAAAAGACGAAGATCACGTCCATTGACGAAGGCTTCGATTTCCTCGGTCAAAACGCCCGGAAGTACGAAGGAAAGCTGTTGATCAAACCATCGAAAACCTCGACTCAGGGACTCCTGGACAAGGTTCGGTTGATCATCGACGCCCACAAAGGCAAATCAGCCGAAAGACTGATCAAGGTACTAAACCCGGTCATCCGTGGCTGGGCCAACTACCACCGCCACAGCGTGTGCGCGCAGACCTTCTATTACATCGACTATGTGATCAGCGGAGCCTTGTTCCGGTGGATACGCAAAAGGAACCAGAATAAATCGAAAAGTTGGATTGTATGGAAACACTTCCGCAGTCCCCTCGACAAATCCGGAACCTTCTGCGCGAAATCGAAAAACAAGAAAGGCCAGACCGTCTACTATCACCTGCAAAAGGCGCTCAACATACCGAGAGCCCTGCATCGAAAGGTAATCGGGAAAGCCCACCCCTACCAACCCGAAAAGGCCGAGTATTTCGCCAAGCGCCAGCTCAAACGTTACCGCACCAAGGGAAGAATGAGCCAGCCGATGCAGTGGATACAAGCCCACCTCGGATTCCAACCATGAAAAGAACAACTGCCGGATCCGCCTCTACTGAGCGGATTCTAGAAATGCTTGAGCCGTGTGAAGGGAAACTTTCACGCACGGTTCTTAGGGAGAACGGGGGCCGCAAGGCCCCCTGACCACCCGGTA

H.ha.F1- fragmented _intron-1187659..1188795

GTGCCCGAGGGCAACACGAAGCACCCGCAATGGCGAGGGTGTGGAGGTCTGGCGGGGTCATCAGGCCGTGGCATGCAGGGAGAGATACGTCGGAGAACTCGGGAAGCCCCGAAGGGCTCCTGTGGTGGTGAAGGCCGGCAAGGGCCTACGGCTATTGAGACACGAAGGGGAAACCTGGAGACGAAGCGCTACCGGACCCGTCGGGTCCGGCGTTGCTACATCCCCAAGGAGGATGGCGGCGAGCGTCCATTGGGGATACCGGCGGTGGAGGACAGGCTGTTGCAAGCGGCCTGTGCTCGGATACTGACCGCCATCTACGAGGCGGACTTTCTGGACGGGAGCTACGGCTACCGGCCAGGGAAGAGCGCTAAGGACGCGGTGGCTGATCTGGGTTCAACCTCTGCACTATGCGCTGGACCTCTGGTTCGAGCAGGTGGTGAAGCCACGTTGTCGAGGACAGGCGCTGCTGGTTCGGTATGCCGATGACTATGTCTGCGCGTTTCAGTTTCAGGAGGATGCCCAGCGCTTCTATCGTGCAGTGCCGCGCCGGCTGGGTCGGTTTGGGCTGCAGGTGGCGCCGGAGAAGACACGGCTGATGCGATTCAGCCGGTTCCATCCGGGGTTGCGGCGACGATTTGGCTTTCTCGGCTTCGAGTTGAACTGGAGCCGGGATCGACGGGGCGAGCTGCGGGTGATGAAGCGCACGGCCCGCAAGAAACTGCAAGCAGCCAAGCGACGGTTGAAGGGCTGGATACGGGCCAACCGGCACCTGCCGGGGCGCGTGTTTATCCAGGAGCTGAATCGTAGACTGGTAGGTCATTACAACTACTTCGGGCTCCGCAGCAATGAGCAGGGTCTAGGGAGCTACCACATCTTCGCCATCCGGTGCGCCTTCAAGTAGCTGAACCGGCGAGGCGGCAAGCGCAGTAGTTTCAACTGGGCGCAATACATTGAGGCCTTGCGGAAGCTGGGAGTGGCACAGCCGCGGATTACGGAGAGGCAACGAGCGCATGGGGTCTTTGCATAAGGGCACGCCCGGCGCGAAGGCGAGTACAACCGAGGAACCGGATGCGGGAAAACCGCACGTCCGGGTCTGTGCGGGGGGGGGCGCCCGGCAACGGGCGTTCCTACCGTGAG

H.ha.F2-fragmented_intron-1196335..1197101

TATCTGCACTACGCGCTGGACCTTTGGTTCGAGCGGGTGGTGAAGCCACGTTGCCGAGGACAGGCGCTGCTGGTTCGGTATGCCGATGACTATGTCTGCGCGTTTCAGTTTCAGGAGGATGCCCAGCGCTTCTATCGTGCAGTGCCGCGCCGGCTGGGTCGGTTTGGGCTGCAGGTGGCGCCGGAGAAGACACGGCTGATGCGATTCAGCCGGTTCCATCCGGGGTTGCGGCGACGATTTGGCTTTCTCGGCTTCGAGTTGTACTGGAGCCGGGATCGGCGGGGCGAGCTGCGGGTGATGAAGCGTACGGTCCGCAAGAAACTGCAAGCAGCCAAGCGGCGGTTGAAGGGCTGGATACGGGCCAACCGGCACCTGCCGGGGCGCGTGTTTATCCAGGAGCTGAATCGTAGACTGGTAGGTCATTACAACTACTTCGGGCTCCGCAGCAATGAGCAGGGTCTAGAGAGCTACTACATCTTCGCCACCCGGTGCGCCTTCAAGTGGCTGAACCGCCGAGGTGGCAAGCGCAGTAGTTTCAACTGGGCGCAATACATTGCGGCGTTGAGGAAGCTGGGAGTGGAGCAGCCGCGGATTACGGAGAGGCAACGAGCGCATGCGGTCTTTGCATAAGGGCACGCCCGTCGCGAAGGCGAGTACAACCGAGGAACCGGATGCGGGAAAACTGCACGTCCGGGTCTGTGCGGGGGCGGCCGGCAACGGGCGTTCCTACCGTGAGATGCAGGCCGGACACCGAGGAT

Fig. S6. Folding of DV and DVI RNA of truncated UHB.F1 within TSL1 metagenomic contig.


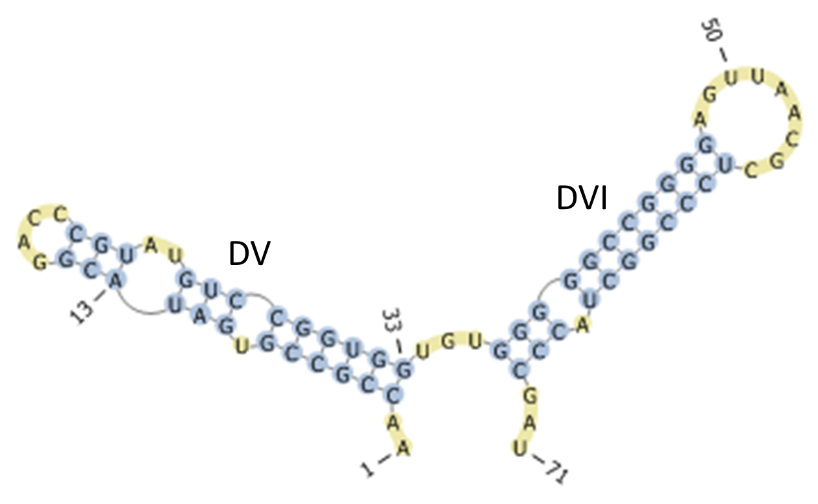


Fig. S7. 5’ exon secondary structure of UHB.I2. *att*C top strand (ts) secondary structure upstream of UHB.I2.


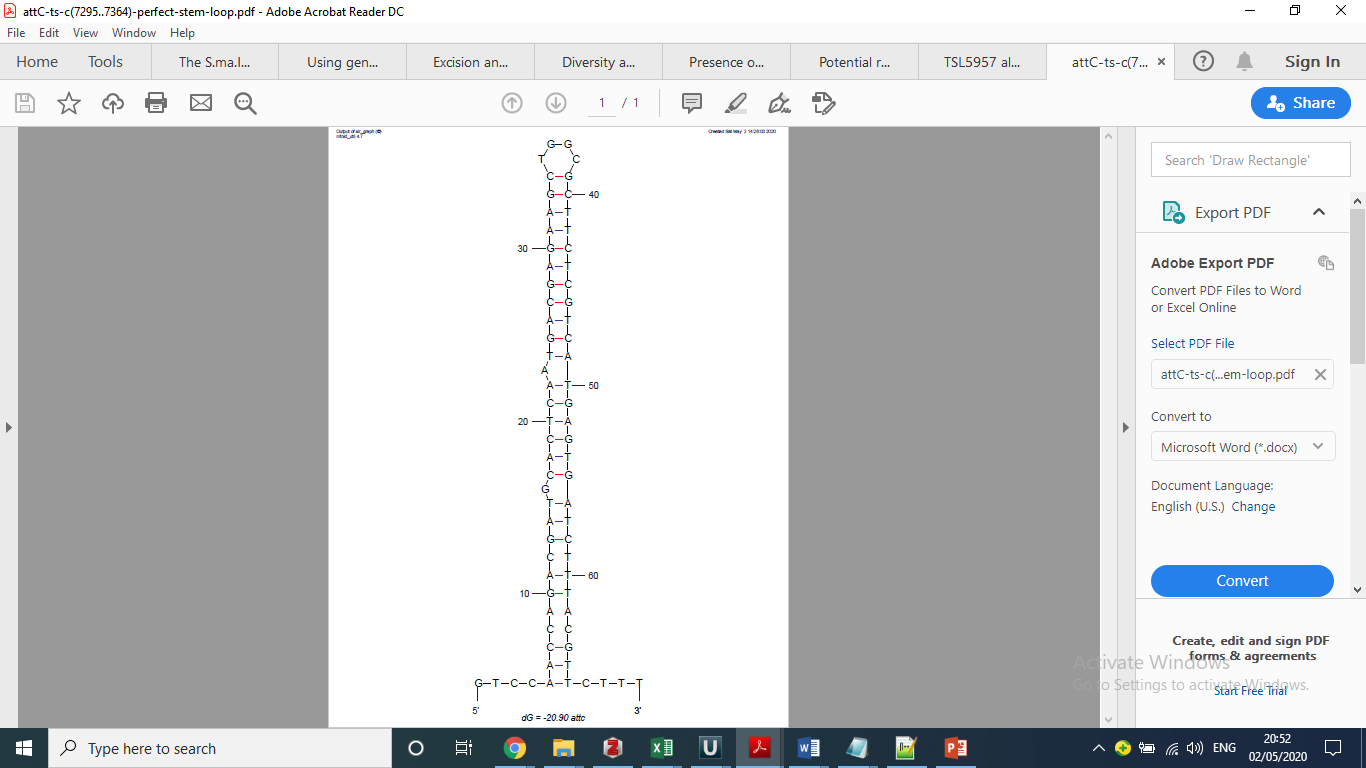


Fig. S8. Folding of DV and DVI RNA of fragmented group II introns identified within a CALIN in *H. halochloris.*


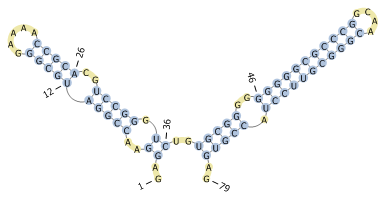

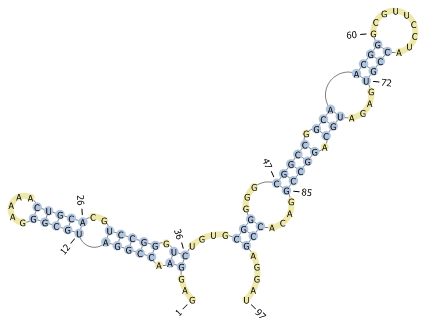


DV

DV

DVI

DVI

**H.ha.F1**

**H.ha.F2**

Fig. S9. Left and right end hairpin structures of IS*Hahl1* compared to IS*CARN6*, both belonging to IS*605* group of IS*200/605* superfamily. A conservation in secondary structure and to a lesser extent in primary structure is shown between left and right ends of both IS elements.


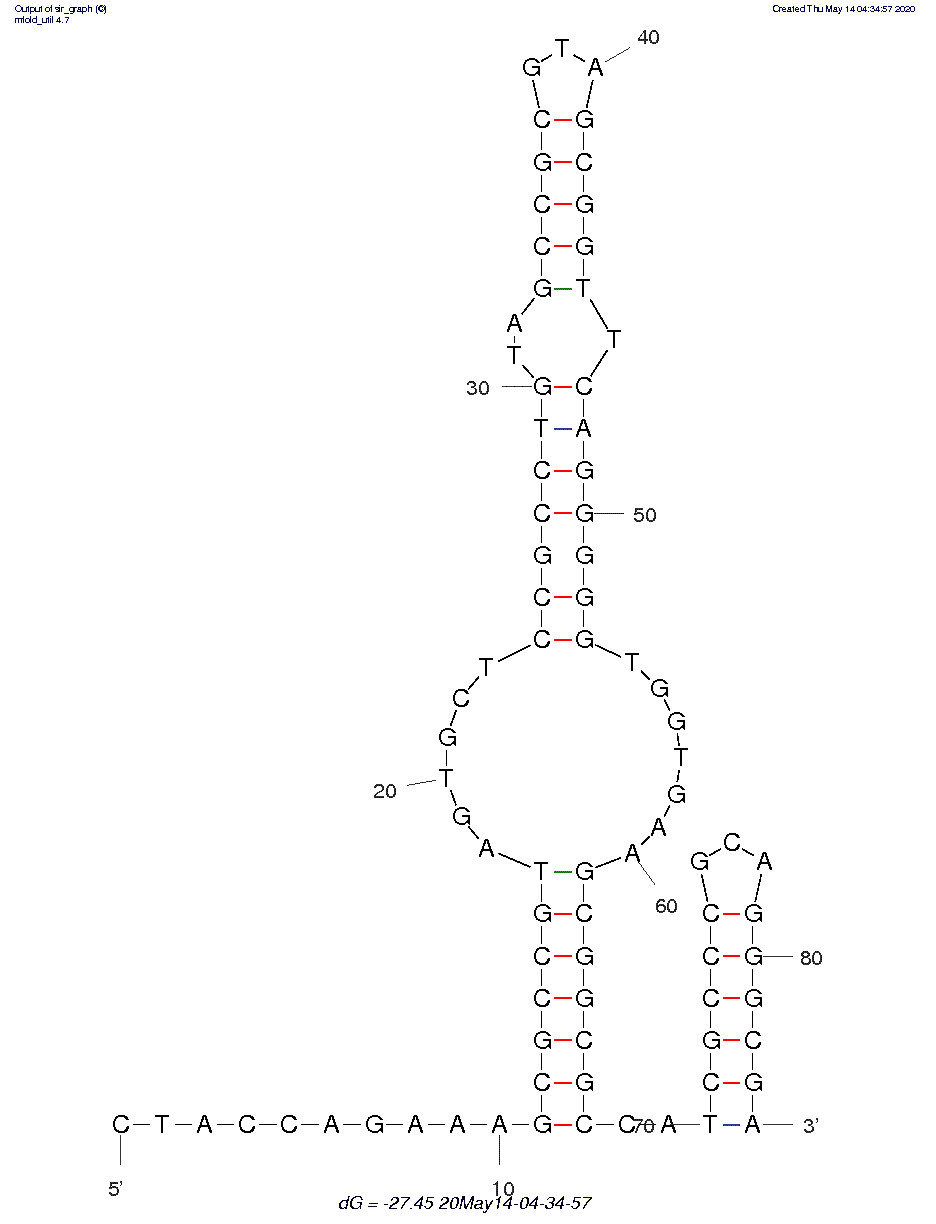

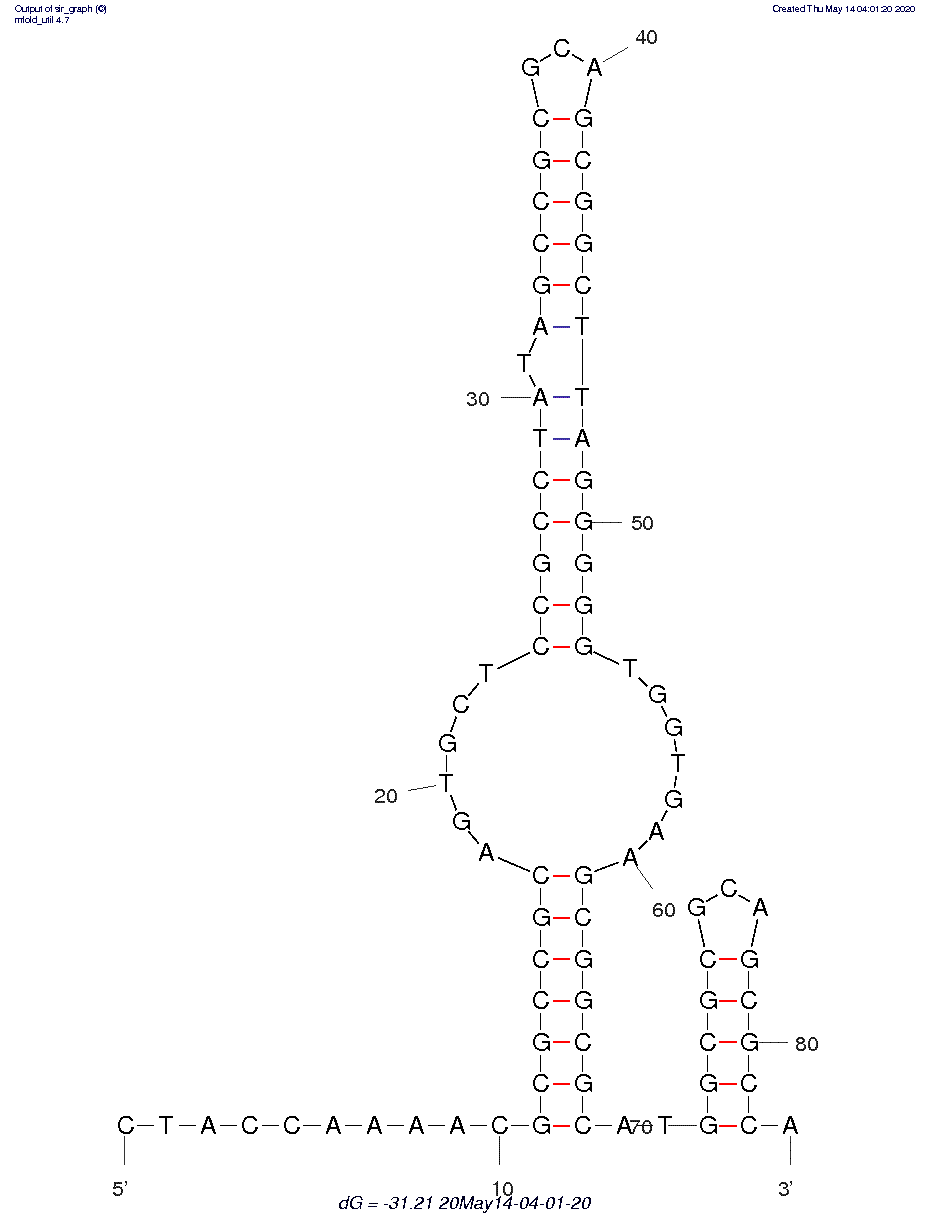

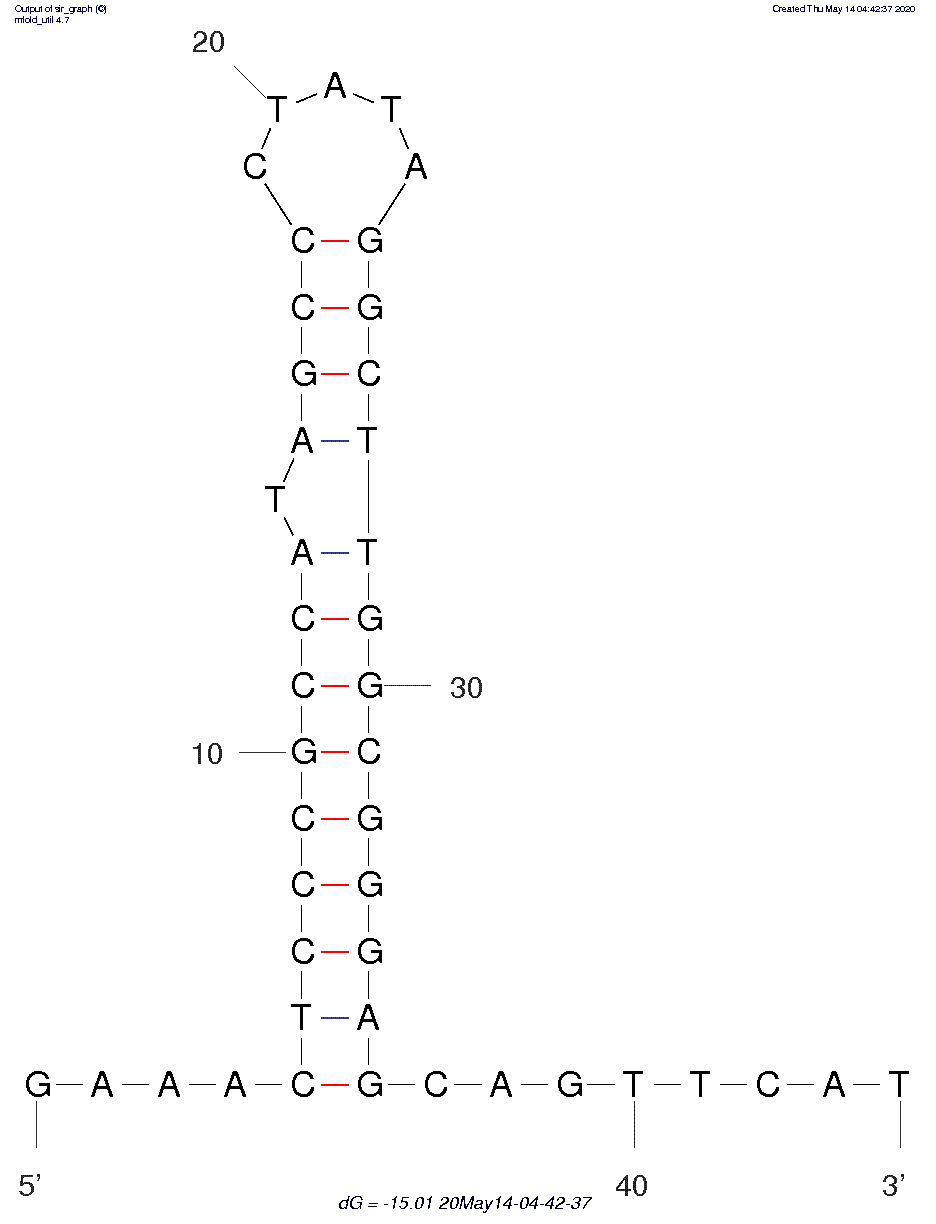

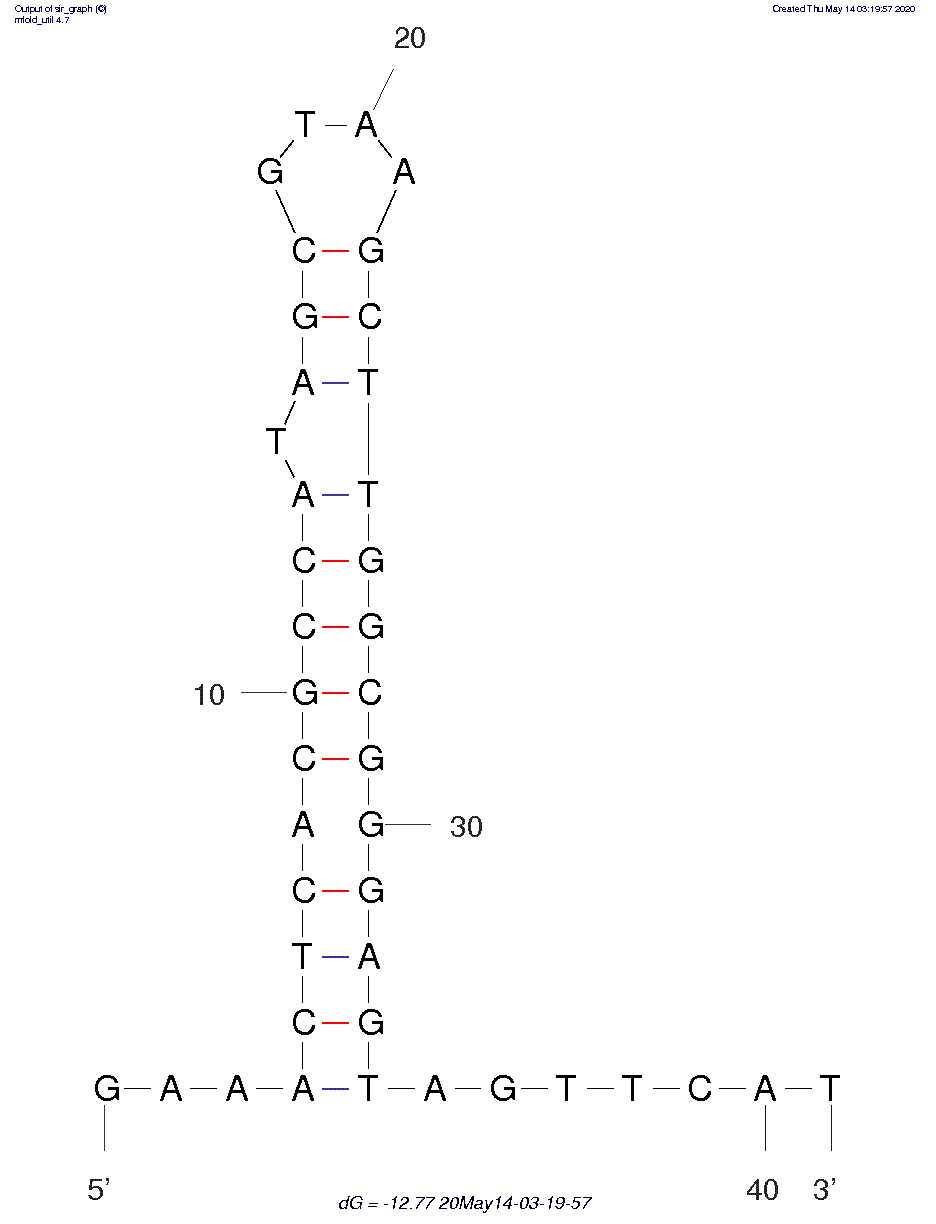


Left end hairpin structure

Right end hairpin structure

IS*Hahl1*

IS*Hahl1*

IS*CARN6*

IS*CARN6*


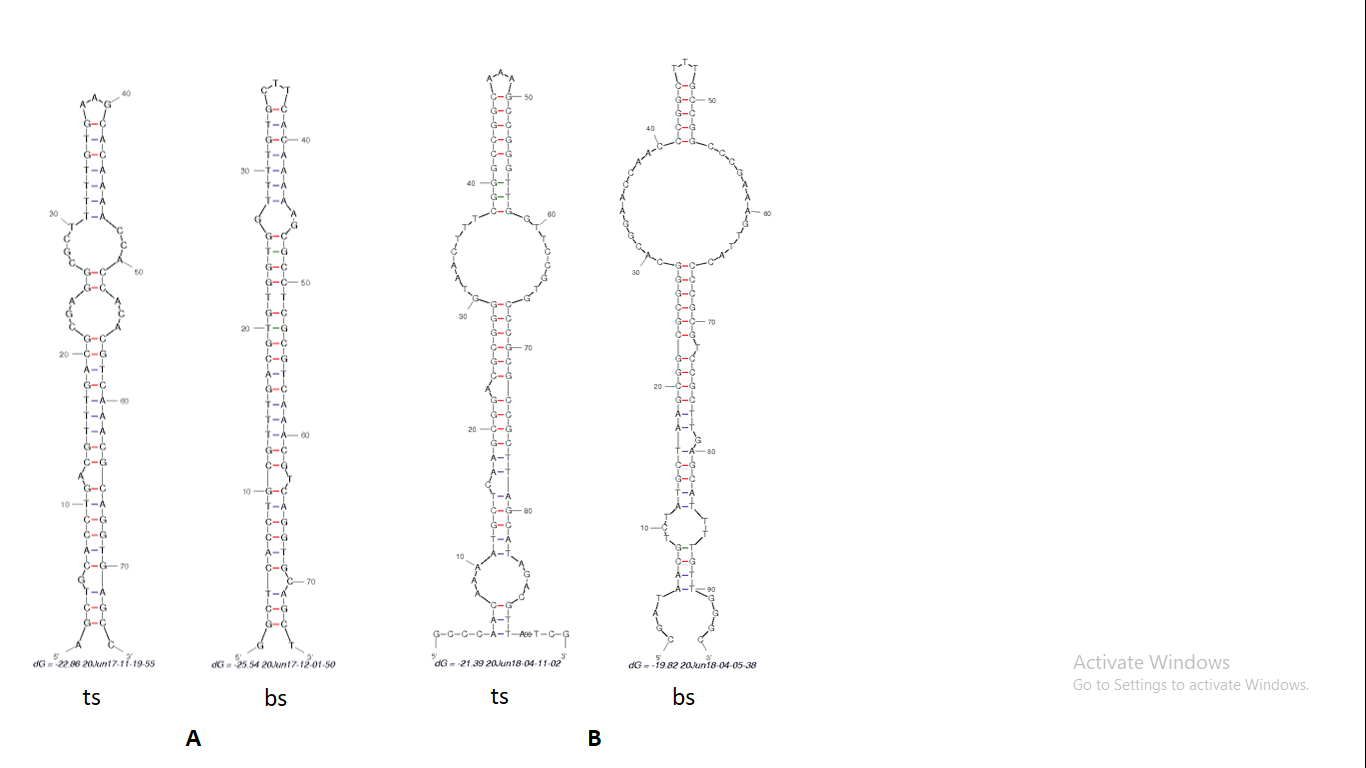
Fig. S10. Secondary structure of putative *att*C sites top strands (ts) and bottom strands (bs) undetected by integron Finder upstream H.ha.F1 and H.ha.F2. A: Atypical *att*C upstream H.ha.F1, B: Putative *att*C upstream H.ha.F2
