# Supplementary material for: The association of group IIB intron with integrons in hypersaline environments
Source: Mob DNA. 2021 Mar 1;12:8. doi: 10.1186/s13100-021-00234-2 (PMC7923331; doi:10.1186/s13100-021-00234-2)
Supplement: Supplementary file 2 — Additional file 2 Table S1. Genetic elements description and position within gene cassette arrays in examined sites. Table S2. Analyzed complete and partial bacterial halophilic genomes. Table S3. Analyzed complete and partial archaeal halophilic genomes. Table S4. Analyzed metagenomic assemblies from different marine, freshwater and hydrothermal vents environments. [file 13100_2021_234_MOESM2_ESM.docx]

Table S1. Genetic elements description and position within gene cassette arrays in examined sites

| **site** | **Genetic element** | **Annotation (description)** | **position** |
| --- | --- | --- | --- |
| TSL1 | Gene cassette ORF | Hypothetical protein | c(9192..9608) |
| TSL1 | *att*C | Integron Finder prediction | c(8999..9068) |
| TSL1 | Gene cassette ORF | Hypothetical protein | c(8709..8987) |
| TSL1 | *att*C | Integron Finder prediction | c(8641..8714) |
| TSL1 | Gene cassette ORF | YoeB-YefM toxin-antitoxin system antitoxin YefM | c(8343..8594) |
| TSL1 | Gene cassette ORF | Txe/YoeB family addiction module toxin | c(8092..8346) |
| TSL1 | *att*C | Integron Finder prediction | c(8025..8092) |
| TSL1 | Gene cassette ORF | Hypothetical protein | c(7726..8007) |
| TSL1 | *att*C | Integron Finder prediction | c(7679..7748) |
| TSL1 | Gene cassette ORF | No significant similarity | c(6893..7672) |
| TSL1 | *att*C | Integron Finder prediction | c(6840..6925) |
| TSL1 | Gene cassette ORF | Serine hydrolase (betalactamase transpeptidase) | c(5676..6734) |
| TSL1 | Gene cassette ORF | Hypothetical protein | c(5183..5524) |
| TSL1 | UHB.F1 | 5’ truncated group IIC intron | 3872..5204 |
| TSL1 | UHB.F1 ORF | Intron encoded protein (group II reverse transcriptase/maturase), 411 aa | 3872..5111 |
| TSL1 | Putative internal promoter | LDF score 1.38,  -10: CGGTAATCT, -35: TCGAGA  no transcription factors binding sites detected | c(4692..4721) |
| TSL1 | Putative internal promoter | LDF score 1.81,  -10: GTTTACCAT, -35: CTGACG  no transcription factors binding sites detected | c(4238..4267) |
| TSL1 | Putative promoter | LDF score 3.04,  -10: TTGTAGTTT, -35: TTGCCA  Binding sites for transcription factors soxS and fis | c(3731..3763) |
| TSL1 | Putative promoter for IEP-ORF | LDF score 2.04,  -10: CGTTGTAAT, -35: TTGTGT  Binding sites for transcription factor rpoD17 | 3675..3701 |
| TSL1 | Putative promoter | LDF score 1.05,  -10: AGGTAGAAA, -35: TTTCCG  Binding sites for transcription factor rpoD15 | c(3399..3426) |
| TSL1 | Putative promoter for IEP-ORF | LDF score 1.0,  -10: TCCGATATT, -35: TTGGCG  Binding sites for transcription factor rpoD16 | 3328..3356 |
| TSL1 | Gene cassette ORF | Hypothetical protein | c(3178..3603) |
| TSL1 | *att*C | Integron Finder prediction | c(2897..3020) |
| TSL1 | Putative promoter for the TA operon | LDF score 0.72,  -10: CGGGAAAAT, -35: GCGCCT  no transcription factors binding sites detected | c(2890..2918) |
| TSL1 | Putative promoter for the TA operon | LDF score 1.79,  -10: CGTTATGAC, -35: TTTCAA  no transcription factors binding sites detected | c(2862..2890) |
| TSL1 | Putative promoter for the toxin ORF | LDF score 3.34,  -10: CAGTATATT, -35: TTGAGG  Binding sites for transcription factor rpoD16 | c(2818..2847) |
| TSL1 | Putative promoter for the toxin ORF | LDF score 4.22,  -10:, ATTGAAAAT, -35: TTGATG  Binding sites for transcription factors rpoD16, ihf and phoB | c(2787..2817) |
| TSL1 | Gene cassette ORF | Antitoxin | c(2618..2839) |
| TSL1 | Gene cassette ORF | RelE/ParE family toxin | c(2371..2631) |
| TSL1 | *att*C | Integron Finder prediction | c(2300..2367) |
| TSL2 | Gene cassette ORF | Hypothetical protein, partial | c(9627..9770) |
| TSL2 | *att*C | Integron Finder prediction | c(9589..9658) |
| TSL2 | Gene cassette ORF | Hypothetical protein | c(9053..9580) |
| TSL2 | *att*C | Integron Finder prediction | c(8978..9047) |
| TSL2 | Gene cassette ORF | Putative GNAT N-acetyltransferase (30% identity) | c(8588..8971) |
| TSL2 | *att*C | Integron Finder prediction | c(8427..8512) |
| TSL2 | Gene cassette ORF | HNH endonuclease | c(7930..8415) |
| TSL2 | *att*C | Integron Finder prediction | c(7890..7959) |
| TSL2 | Gene cassette ORF | PH domain-containing protein | c(7420..7884) |
| TSL2 | *att*C and stem loop | Integron Finder prediction | c(7295..7364) |
| TSL2 | Putative internal IEP-ORF promoter | LDF score 3.92,  -10: TGATATAAT, -35: CTGATT  Binding sites for transcription factor rpoD16 | c(7246..7271) |
| TSL2 | UHB.I2 | IIB1 group II Intron | c(5096.. 7296) |
| TSL2 | Putative internal IEP-ORF promoter | LDF score 1.04,  -10: TGATAAACC, -35: TTTCTT  Binding sites for transcription factor crp | c(6745..6771) |
| TSL2 | IEP | Chloroplast-like 1(CL1) IEP, 500 aa | c(5223..6725) |
| TSL2 | Putative internal promoter | LDF score 2.69,  -10: GCGTAGAAT, -35: CTACCG  Binding sites for transcription factor narL | c(6397..6423) |
| TSL2 | Putative internal promoter | LDF score 1.27,  -10: TGTTAACGT, -35: GTCCCG  Binding sites for transcription factor rpoD16 | c(5932..5960) |
| TSL2 | Putative internal promoter | LDF score 1.08,  -10: GTCTACTAT, -35: TCGAAA  no transcription factors binding sites detected | c(5406..5438) |
| TSL2 | Gene cassette ORF | Hypothetical protein | c(4525..5097) |
| TSL2 | Putative promoter for TA operon | LDF score 2.17,  -10: CGGAATATT, -35: GTGATG  Binding sites for transcription factors crp and rpoD19 | c(4709..4737) |
| TSL2 | *att*C | Integron Finder prediction | c(4463..4591) |
| TSL2 | Putative promoter for the toxin gene | LDF score 0.97,  -10: TACTGTAAT, -35: ATGCTA  no transcription factors binding sites detected | c(4405..4435) |
| TSL2 | ORF downstream the gene cassette array | ParD-like antitoxin | c(4200..4418) |
| TSL2 | ORF downstream the gene cassette array | RelE/ParE family toxin | c(3923..4213) |
| *Halorhodospira halochloris* DSM 1059 | IS*200/605* element | 70% coverage to IS*Hahl*1 with 98% identity. A deletion in the middle, thus missing 5’end of both *tnp*A and *tnp*B genes. Y1 transposase domain in *tnp*A is complete | 449664..450944 |
| *Halorhodospira halochloris* DSM 1059 | IS*200/605* element | 80% coverage to IS*Hahl*1 with 98% identity. A deletion in the 5’ end, thus having a truncated *tnp*A gene and a complete *tnp*B gene. | 460538..461995 |
| *Halorhodospira halochloris* DSM 1059 | IS*200/605* element | 57% identity to IS*Hahl*1. Frameshifts in both *tnp*A and *tnp*B genes most probably rendering them inactive | c(690007..691779) |
| *Halorhodospira halochloris* DSM 1059 | Partial IS*200/605* element | 17% coverage to IS*Hahl*1 with 95% identity. No transposase genes detected | 767946..769352 |
| *Halorhodospira halochloris* DSM 1059 | Putative CALIN promoter | LDF score 2.52,  -10: CCTTATAAA, -35: CTGCTT  Binding sites for transcription factors metR, rpoD17, rpoD16 | 1184472..1184504 |
| *Halorhodospira halochloris* DSM 1059 | Putative CALIN promoter | LDF score 1.22,  -10: CAGTATCCT, -35: CTGCGA  Binding sites for transcription factor rpoD16 | 1184783..1184809 |
| *Halorhodospira halochloris* DSM 1059 | Gene cassette ORF | Hypothetical protein | 1184958..1185458 |
| *Halorhodospira halochloris* DSM 1059 | *att*C | Integron Finder prediction | 1185442..1185516 |
| *Halorhodospira halochloris* DSM 1059 | Putative promoter for TA operon | LDF score 3.41,  -10: GCATACAAT, -35: TTGACC  Binding sites for transcription factor rpoD18 | 1185541..1185569 |
| *Halorhodospira halochloris* DSM 1059 | Gene cassette ORF | BrnT family toxin | 1185576..1185863 |
| *Halorhodospira halochloris* DSM 1059 | Gene cassette ORF | BrnA family antitoxin | 1185860..1186075 |
| *Halorhodospira halochloris* DSM 1059 | *att*C | Integron Finder prediction | 1186078..1186137 |
| *Halorhodospira halochloris* DSM 1059 | Gene cassette ORF | NgoFVII family restriction endonuclease | 1186155..1186583 |
| *Halorhodospira halochloris* DSM 1059 | Gene cassette ORF | Hypothetical protein | 1186587..1187339 |
| *Halorhodospira halochloris* DSM 1059 | *att*C-like | CAC and GTG instead of the conserved triad (AAC and GTT) in the R box, no unpaired spacer between R & L boxes, | 1187352..1187425 |
| *Halorhodospira halochloris* DSM 1059 | Putative promoter for IEP ORF | LDF score 0.9,  -10: GGTTAAGCG, -35: GTGAGG  Binding sites for transcription factors rpoD18 & ihf | 1187532..1187562 |
| *Halorhodospira halochloris* DSM 1059 | H.ha.F1 | 5’ truncated IIB group II Intron | 1187659..1188795 |
| *Halorhodospira halochloris* DSM 1059 | IEP | Bacterial class E IEP, 342 aa, internal deletion causing a frameshift at 133 and an internal stop at 300 | 1187659..1188685 |
| *Halorhodospira halochloris* DSM 1059 | Putative internal promoter | LDF score 1.05,  -10: TCGTAGACT, -35: TTTATC  no transcription factors binding sites detected | 1188443..1188468 |
| *Halorhodospira halochloris* DSM 1059 | Putative internal promoter | LDF score 0.91,  -10: CGGTATGCC, -35: TTGTCG  no transcription factors binding sites detected | 1188106..1188139 |
| *Halorhodospira halochloris* DSM 1059 | Putative promoter for the TA system | LDF score 2.38,  -10: CGTTATTAA, -35: TTGCCA  no transcription factors binding sites detected | 1188819..1188846 |
| *Halorhodospira halochloris* DSM 1059 | Gene cassette ORF | RelE/ParE family toxin | 1188869..1189147 |
| *Halorhodospira halochloris* DSM 1059 | Gene cassette ORF | HigA family antitoxin | 1189158..118472 |
| *Halorhodospira halochloris* DSM 1059 | *att*C | Integron Finder prediction | 1189467..1189526 |
| *Halorhodospira halochloris* DSM 1059 | Gene cassette ORF | DUF1643 domain-containing protein | 1189540..1190004 |
| *Halorhodospira halochloris* DSM 1059 | *att*C | Integron Finder prediction | 1190007..1190078 |
| *Halorhodospira halochloris* DSM 1059 | Gene cassette ORF | DUF3800 domain-containing protein | 1190085..1190897 |
| *Halorhodospira halochloris* DSM 1059 | *att*C | Integron Finder prediction | 1190899..1190970 |
| *Halorhodospira halochloris* DSM 1059 | Gene cassette ORF | SIR2 family protein | 1191028..1192185 |
| *Halorhodospira halochloris* DSM 1059 | Gene cassette ORF | DUF4160 domain-containing protein | 1192152..1192376 |
| *Halorhodospira halochloris* DSM 1059 | Gene cassette ORF | DUF2442 domain-containing protein | 1192479..1192751 |
| *Halorhodospira halochloris* DSM 1059 | Gene cassette ORF | HNH endonuclease | 1192791..1193168 |
| *Halorhodospira halochloris* DSM 1059 | Putative promoter for TA operon within upstream ORF | LDF score 2.9,  -10: GAGTATAAG, -35: GTCATA  Binding sites for transcription factors rpoD16, rpoD15 & purR | 1193084..1193116 |
| *Halorhodospira halochloris* DSM 1059 | Gene cassette ORF | antitoxin | 1193223..1193453 |
| *Halorhodospira halochloris* DSM 1059 | Gene cassette ORF | RelE/ParE family toxin | 1193453..1193749 |
| *Halorhodospira halochloris* DSM 1059 | *att*C | Integron Finder prediction | 1193745..1193828 |
| *Halorhodospira halochloris* DSM 1059 | Gene cassette ORF | DUF4160 domain-containing protein | 1193885..1194151 |
| *Halorhodospira halochloris* DSM 1059 | Gene cassette ORF | DUF2442 domain-containing protein | 1194160..1194408 |
| *Halorhodospira halochloris* DSM 1059 | *att*C | Integron Finder prediction | 1194472..1194535 |
| *Halorhodospira halochloris* DSM 1059 | Putative promoter for TA operon within upstream gene cassette ORF | LDF score 0.24,  -10: TCGTACTTT, -35 TTTTTA  Binding sites for transcription factor rpoD16 | 1194272..1194303 |
| *Halorhodospira halochloris* DSM 1059 | Gene cassette ORF | BrnT family toxin | 1194537..1194854 |
| *Halorhodospira halochloris* DSM 1059 | Gene cassette ORF | BrnA family antitoxin | 1194851..1195123 |
| *Halorhodospira halochloris* DSM 1059 | Putative promoter for TA operon | LDF score 3.13,  -10: CGGCATTTT, -35: TTGACA  Binding sites for transcription factors rpoD16 & rpoD17 | 1195221..1195254 |
| *Halorhodospira halochloris* DSM 1059 | Gene cassette ORF | BrnT family toxin | 1195502..1195870 |
| *Halorhodospira halochloris* DSM 1059 | Putative promoter for antitoxin gene within toxin ORF | LDF score 1.78,  -10: ATGCATACT, -35: TTGGCT  no transcription factors binding sites detected | 1195568..1195596 |
| *Halorhodospira halochloris* DSM 1059 | Gene cassette ORF | BrnA family antitoxin | 1195863..1196114 |
| *Halorhodospira halochloris* DSM 1059 | *att*C | Predicted by bs folding using MFOLD | 1196117..1196210 |
| *Halorhodospira halochloris* DSM 1059 | Putative promoter within detected attC site | LDF score 1.16,  -10: GCTTAGCAT, -35: TTGGTT  no transcription factors binding sites detected | 1196172..1196199 |
| *Halorhodospira halochloris* DSM 1059 | H.ha.F2 | 5’ truncated IIB group II Intron | 1196335..1197101 |
| *Halorhodospira halochloris* DSM 1059 | IEP | Bacterial class E IEP, 210 aa, 5’ deletion | 1196335..1196964 |
| *Halorhodospira halochloris* DSM 1059 | Putative internal promoter | LDF score 1.20,  -10: CGGTATGCC, -35: TTGCCG  no transcription factors binding sites detected | 1196390..1196418 |
| *Halorhodospira halochloris* DSM 1059 | Putative internal promoter | LDF score 1.05,  -10: TCGTAGACT, -35: TTTATC  no transcription factors binding sites detected | 1196722..1196747 |
| *Halorhodospira halochloris* DSM 1059 | Gene cassette ORF-frame shift | HicA family toxin-frame-shift due to 1 nucleotide deletion at 1197266 position | 1197111.. 1197370 |
| *Halorhodospira halochloris* DSM 1059 | Putative promoter for antitoxin gene within upstream toxin gene and 3’ end of the intron | LDF score 0.89,  -10: TGAGAAAAT, -35: TTACAA  no transcription factors binding sites detected | 1197092..1197120 |
| *Halorhodospira halochloris* DSM 1059 | Putative promoter for antitoxin gene within upstream toxin gene | LDF score 2.37,  -10: GGCTAGGAT, -35: TTGTCA  no transcription factors binding sites detected | 1197185..1197210 |
| *Halorhodospira halochloris* DSM 1059 | Gene cassette ORF | HicB family antitoxin | 1197360..1197572 |
| *Halorhodospira halochloris* DSM 1059 | *att*C | Integron Finder prediction | 1197567..1197626 |
| *Halorhodospira halochloris* DSM 1059 | IS*Hahl*1 | IS200/605 family (IS605 group) insertion sequence | 1197652..1199464 |
| *Halorhodospira halochloris* DSM 1059 | IS*Hahl*1-LE | IS left end forming hairpin structure | 1197652..1197730 |
| *Halorhodospira halochloris* DSM 1059 | TnpA | ISHahl1 TnpA (transposase) | c(1197731..1198045) |
| *Halorhodospira halochloris* DSM 1059 | TnpB | ISHahl1 TnpB (accessory protein) | 1198170..1199444 |
| *Halorhodospira halochloris* DSM 1059 | IS*Hahl*1-RE | IS right end forming hairpin structure | 1199445..1199464 |
| *Halorhodospira halochloris* DSM 1059 | IS*Hahl*1 isoform | IS200/605 family (IS605 group) insertion sequence | 1269635..1271447 |
| *Halorhodospira halochloris* DSM 1059 | IS*200/605* element | 57% coverage to IS*Hahl*1 with 97% identity. A deletion in the middle, thus missing N-termini of both *tnp*A and *tnp*B genes. | c(1472999..1474045) |
| *Halorhodospira halochloris* DSM 1059 | *Ori*C | Predicted OriC by γBORIS | 2787842..2789091 |

Table S2. Analyzed complete and partial bacterial halophilic genomes

| bacterial analysed genomes | genome size | sequencing status | genome or WGS accession number | plasmids accession numbers if present (for complete genomes) |
| --- | --- | --- | --- | --- |
| *Acetohalobium arabaticum* DSM 5501 | 2.4696 | complete | NC_014378.1 | - |
| *Halothece* sp. PCC 7418 | 4.17917 | complete | NC_019779.1 | - |
| *Cellulosimicrobium cellulans* PSBB019 | 4.79986 | complete | NZ_CP021383.1 | - |
| *Desulfohalobium retbaense* DSM 5692 | 2.90957 | complete | NC_013223.1 | NC_013224.1 |
| *Chromohalobacter salexigens* DSM 3043 | 3.66514 | complete | NC_007963.1 | - |
| *Halorhodospira halophila* SL1 | 2.67845 | complete | NC_008789.1 | - |
| *Halorhodospira halochloris* DSM 1059 | 2.83456 | complete | NZ_AP017372.2 | - |
| *Halanaerobium hydrogeniformans* | 2.61312 | complete | NC_014654.1 | - |
| *Halanaerobium praevalens* DSM 2228 | 2.30926 | complete | NC_017455.1 | - |
| *Halobacillus halophilus* DSM 2266 | 4.17177 | complete | NC_017668.1 | NC_017670.1, NC_017669.1 |
| *Halobacteroides halobius* DSM 5150 | 2.64926 | complete | NC_019978.1 | - |
| *Halomonas elongata* DSM 2581 | 4.06182 | complete | NC_014532.2 | - |
| *Halomonas titanicae* ANRCS81 | 5.33979 | complete | NZ_CP039374.1 | - |
| *Halothermothrix orenii* H 168 | 2.57815 | complete | NC_011899.1 | - |
| *Marinobacter hydrocarbonoclasticus* ATCC 49840 | 3.98677 | complete | NC_017067.1 | - |
| *Marinobacter hydrocarbonoclasticus* VT8 | 4.77976 | complete | NC_008740.1 | NC_008738.1, NC_008739.1 |
| *Natranaerobius thermophilus* JW/NM-WN-LF | 3.19145 | complete | NC_010718.1 | NC_010715.1, NC_010724.1 |
| *Nitrosococcus halophilus* Nc 4 | 4.14526 | complete | NC_013960.1 | NC_013958.1 |
| *Nodularia spumigena* CCY9414 | 5.35144 | complete | NZ_CP007203.1 | - |
| *Nodularia spumigena* UHCC 0039 | 5.38661 | complete | NZ_CP020114.1 | NZ_CP020115.1 |
| *Oceanobacillus iheyensis* HTE831 | 3.63053 | complete | NC_004193.1 | - |
| *Oceanobacillus iheyensis* CHQ24 | 3.86062 | complete | NZ_CP020357.1 | - |
| *Salinibacter ruber* DSM 13855 | 3.76289 | complete | NC_007677.1 | NC_007678.1 |
| *Spiribacter salinus* M19-40 | 2.88033 | complete | NC_021291.1 | - |
| *Ectothiorhodospira haloalkaliphila* A | 3.46013 | partial | NZ_CP007268.1 | - |
| *Alteribacillus bidgolensis* DSM 25260 | 4.70318 | partial | NJAU01 | - |
| *Alteribacillus bidgolensis* P4B,CCM 7963,CECT 7998,DSM 25260,IBRC-M 10614,KCTC 13821 genome assembly | 4.464 | partial | FNDU01 | - |
| *Alteribacillus persepolensis* DSM 21632 | 3.6191 | partial | NZ_FNDK01000000 | - |
| *Chlorogloea fritschii* PCC 6912 | 7.75174 | partial | RSCJ01 | - |
| *Chromohalobacter japonicus* CJ | 3.37628 | partial | NZ_CDGZ01000000 | - |
| *Chromohalobacter japonicus* SMB17 | 3.76792 | partial | MSDQ01 | - |
| *Desulfovibrio oxyclinae* DSM 11498 | 3.32458 | partial | NZ_AQXE01000000 | - |
| *Ectothiorhodospira mobilis* DSM 4180 | 2.62495 | partial | NZ_FOUO00000000.1 | - |
| *Halarsenatibacter silvermanii* SLAS-1 | 2.71864 | partial | NZ_FNGO00000000.1 | - |
| *Halobacillus aidingensis* CGMCC 1.3703 | 4.19184 | partial | NZ_FNIZ00000000.1 | - |
| *Halobacillus alkaliphilus* FP5 | 4.09253 | partial | NZ_FOOG00000000.1 | - |
| *Halobacillus dabanensis* CGMCC 1.3704 | 4.11984 | partial | FOSB01 | - |
| *Halobacillus dabanensis* HD-02 | 4.10233 | partial | CCDH01 | - |
| *Halobacillus trueperi* SS1 | 4.25856 | partial | QTLC01 | - |
| *Halomonas arcis* CGMCC 1.6494 | 4.14213 | partial | NZ_FNII00000000.1 | - |
| *Halomonas halodenitrificans* DSM 735 | 3.46409 | partial | NZ_JHVH00000000.1 | - |
| *Halomonas meridiana* ACAM 246 | 3.84974 | partial | FSQY01 | - |
| *Halomonas saccharevitans* CGMCC 1.6493 | 3.68129 | partial | NZ_FPAQ00000000.1 | - |
| *Halomonas subterranea* CGMCC 1.6495 | 3.7342 | partial | NZ_FOGS00000000.1 | - |
| *Halonatronum saccharophilum* DSM 13868 | 2.88452 | partial | NZ_AZYG00000000.1 | - |
| *Microcoleus chthonoplastes* PCC 7420 | 8.67904 | partial | ABRS01 | - |
| *Nocardiopsis halotolerans* DSM 44410 | 6.26393 | partial | NZ_ANAX00000000.1 | - |
| *Pontibacillus halophilus* JSM 076056 = DSM 19796 | 3.6014 | partial | AULI01 | - |
| *Saccharomonospora halophila* 8 | 3.68502 | partial | AICX01 | - |
| *Salinovibrio costicola* ATCC 33508 = LMG 11651 | 4.78167 | partial | ASAI01 | - |
| *Salinovibrio costicola* PRJEB21454 | 3.32115 | partial | FYET01 | - |
| *Salisaeta longa* DSM 21114 | 3.39902 | partial | NZ_ATTH00000000.1 | - |
| *Sediminibacillus halophilus* CGMCC 1.6199 | 4.147699 | partial | NZ_FNHF00000000.1 | - |
| *Sediminibacillus halophilus* NSP9.3 | 3.986 | partial | AWXX01 | - |
| *Selenihalanaerobacter shriftii* ATCC BAA-73 | 2.84058 | partial | NZ_FUWM00000000.1 | - |
| *Spirulina subsalsa* PCC 9445 | 5.3236 | partial | NZ_ALVR00000000.1 | - |
| *Streptomyces radiopugnans* CGMCC 4.3519 | 6.06712 | partial | NZ_FOET00000000.1 | - |
| *Thalassobacillus cyri* CCM7597 | 4.30083 | partial | NZ_FNQR00000000.1 | - |

Table S3. Analyzed complete and partial archaeal halophilic genomes

| archaeal analysed genomes | genome size (Mb) | sequencing status | genome or WGS  accession number | plasmids accession numbers if present (for complete genomes) |
| --- | --- | --- | --- | --- |
| *Halalkalicoccus jeotgali* B3 | 3.69865 | complete | NC_014297.1 | NC_014298.1, NC_014299.1, NC_014300.1 , NC_014300.1, NC_014302.1, NC_014303.1 |
| *Haloarcula hispanica* ATCC 33960 | 3.89 | complete | NC_015948.1 , NC_015943.1 | NC_015944.1 |
| *Haloarcula marismortui* ATCC 43049 | 4.27464 | complete | NC_006396.1 , NC_006397.1 | NC_006389.1, NC_006389.1, NC_006389.1, NC_006392.1, NC_006392.1, NC_006393.1 , NC_006394.1, NC_006395.1 |
| *Haloarcula* sp CBA1115 | 4.22505 | complete | NZ_CP010529.1 | , NZ_CP010531.1, NZ_CP010532.1, NZ_CP010533.1, NZ_CP010534.1NZ_CP010530.1 |
| *Halobacterium salinarum* NRC-1 | 2.57101 | complete | NC_002607.1 | NC_001869.1, NC_002608.1 |
| *Halobacterium walsbyi* C23 | 3.36799 | complete | NC_017459.1 | NC_017460.1, NC_017460.1, NC_017457.1 |
| *Haloferax gibbonsii* ARA6 | 3.91845 | complete | NZ_CP011947.1 | NZ_CP011948.1, NZ_CP011949.1, NZ_CP011950.1, NZ_CP011951.1 |
| *Haloferax mediterranei* ATCC33500 | 3.90471 | complete | NC_017941.2 | NC_017942.1, NC_017943.1, NC_017944.1 |
| *Haloferax volcanii* DS2 | 4.0129 | complete | NC_013967.1 | NC_013968.1 , NC_013965.1, NC_013964.1, NC_013966.1 |
| *Halogeometricum borinquense* DSM 11551 | 3.94447 | complete | NC_014729.1 | NC_014735.1, NC_014731.1, NC_014736.1, NC_014732.1, NC_014732.1, NC_014737.1 |
| *Halomicrobium mukohataei* DSM 12286 | 3.33235 | complete | NC_013202.1 | NC_013201.1 |
| *Halopiger xanaduensis* SH-6(T) | 4.35527 | complete | CP002839.1 | CP002840.1, CP002841.1, CP002842.1 |
| *Halorhabdus utahensis* DSM 12940 | 3.116795 | complete | CP001687.1 | - |
| *Halorubrum lacusprofundi* ATCC 49239 | 3.69258 | complete | NC_012029.1, NC_012028.1 | NC_012030.1 |
| *Haloterrigena turkmenica* DSM 5511 | 5.44078 | complete | NC_013743.1 | NC_013744.1, NC_013745.1, NC_013746.1, NC_013747.1, NC_013748.1, NC_013749.1 |
| *Halovivax ruber* XH-70 | 3.22388 | complete | NC_019964.1 | - |
| *Mathanohalobium evestigatum* Z-7303 | 2.406232 | complete | NC_014253.1 | NC_014254.1 |
| *Methanohalophilus halophilus* Z-7982 | 2.02296 | complete | NZ_CP017921.1 | - |
| *Methanohalophilus mahii* DSM 5219 | 2.012424 | complete | NC_014002.1 | - |
| *Methanosalsum zhilinae* DSM 4017 | 2.138444 | complete | NC_015676.1 | - |
| *Methanosarcina acetivorans* C2A | 5.75149 | complete | AE010299.1 | - |
| *Natrialba magadii* ATCC 43099 | 4.44364 | complete | NC_013922.1 | NC_013923.1 , NC_013924.1, NC_013925.1 |
| *Natronobacterium gregoryi* SP2 | 3.78836 | complete | NC_019792.1 | - |
| *Natronococcus occultus* SP4 | 4.314118 | complete | NC_019974.1 | NC_019975.1, NC_019976.1 |
| *Natronomonas pharaonis* DSM 2160 | 2.7497 | complete | NC_007426.1 | NC_007427.1, NC_007428.1 |
| *Methanohalophilus portucalensis* FDF-1T | 2.08498 | partial | NZ_CP017881.1 | - |
| *Haloarcula amylolytica* JCM 13557 | 4.22542 | partial | NZ_AOLW00000000.1 | - |
| *Haloarcula argentinensis* DSM 12282 | 4.14711 | partial | NZ_AOLX00000000.1 | - |
| *Haloarcula japonica* DSM 6131 | 4.28036 | partial | NZ_AOLY00000000.1 | - |
| *Haloarcula vallismortis* ATCC 29715 | 3.90992 | partial | NZ_AOLQ00000000.1 | - |
| *Halobacterium jilantaiense* CGMCC 1.5337 | 2.95279 | partial | NZ_FOJA00000000.1 | - |
| *Halobaculum gomorrense* DSM 9297 | 3.20825 | partial | NZ_FQWV00000000.1 | - |
| *Halococcus morrhuae* DSM 1307 | 2.99156 | partial | NZ_AOMC00000000.1 | - |
| *Halococcus saccharolyticus* DSM 5350 | 3.4497 | partial | NZ_AOMD00000000.1 | - |
| *Halococcus sulifodinae* DSM 8989 | 4.19978 | partial | NZ_AOME00000000.1 | - |
| *Haloferax denitrificans* ATCC 35960 | 3.82597 | partial | NZ_AOLP00000000.1 | - |
| *Haloferax elongans* ATCC BAA-1513 | 3.95214 | partial | NZ_AOLK00000000.1 | - |
| *Haloferax mucosum* ATCC BAA-1512 | 3.36898 | partial | NZ_AOLN00000000.1 | - |
| *Haloferax sulfurifontis* ATCC BAA-897 | 3.81243 | partial | NZ_AOLM00000000.1 | - |
| *Halorubrum coriense* DSM 10284 | 3.64531 | partial | NZ_AOJL00000000.1 | - |
| *Halorubrum distributum* JCM 10118 | 3.30613 | partial | AOJN01 | - |
| *Halorubrum distributum* JCM 9100 | 3.30737 | partial | AOJM01 | - |
| *Halorubrum distributum* E8 | 2.25364 | partial | NHPH01 | - |
| *Halorubrum saccharovorum* DSM 1137 | 3.35304 | partial | AOJE01 | - |
| *Halorubrum sodomense* RD 26 | 3.03055 | partial | NZ_FOYN00000000.1 | - |
| *Halosimplex carlsbadense* 2-9-1 | 4.69489 | partial | NZ_AOIU00000000.1 | - |
| *Natronococcus amylolyticus* DSM 10524 | 4.41653 | partial | NZ_AOIB00000000.1 | - |

Table S4. Analyzed metagenomic assemblies from different marine, freshwater and hydrothermal vents environments

|  | Site | Description | Assembly Accession number or reference | Total assembled sequence length | Number of contigs |
| --- | --- | --- | --- | --- | --- |
| Marine | ADR | North Adriatic Sea, Italy, depth 1m | GCA_900205615.1 | 24428552 | 29430 |
|  | ARC | Arctic Ocean, station 54, depth 40.3m | GCA_900247125.1 | 6551393 | 10186 |
|  | PAC | Pacific Ocean, depth 100m | GCA_002896035.2 | 201472418 | 193946 |
|  | Red10 | Red Sea water column Station 192 - depth 10m | GCA_001626065.1 | 97729439 | 57007 scaffolds |
|  | Red25 | Red Sea water column Station 192 - depth 25m | GCA_001629045.1 | 57846509 | 34483 scaffolds |
|  | Red50 | Red Sea water column Station 192 - depth 50m | GCA_001629095.1 | 86416103 | 47563 scaffolds |
|  | Red100 | Red Sea water column Station 192 - depth 100m | GCA_001629115.1 | 50269729 | 34015 scaffolds |
|  | Red200 | Red Sea water column Station 192 - depth 200m | GCA_001629075.1 | 45247809 | 30314 scaffolds |
|  | Red500 | Red Sea water column Station 192 - depth 500m | GCA_001629135.1 | 72981833 | 44066 scaffolds |
|  | SOCo | Metagenomic co-assembly of South Ocean 3 biosamples: SAMEA2621487, SAMEA2621509, SAMEA2621536, depth 5m | GCA_001757065.1 | 185494017 | 19160 |
|  | TIB | Trindade and Martin Vaz Islands, Eastern Brazil, depth 5m | GCA_001371195.1 | 110278656 | 116750 |
|  | WIO | Western Indian Ocean, Fiji islands and Western and Northern Madagascar, depth 5m | GCA_001370375.1 | 199208958 | 216738 |
|  | CIOI | Central Indian Ocean Islands, depth 5m | GCA_001370295.1 | 70690895 | 62491 |
|  | WSIS | West and South Indian Shelf, depth 5m | GCA_001370155.1 | 53135041 | 47352 |
|  | MED | Mediterranean Sea (Tunisian Plateau/Gulf of Sidra & Ionian Sea), depth 5m | GCA_001369555.1 | 99812943 | 73799 |
|  | ATII 50 | Atlantis II 50 m water column, red Sea | [1], [2] | 53647835 | 78510 |
|  | ATII 200 | Atlantis II 200 m water column, red Sea | [1], [2] | 49971663 | 72359 |
|  | ATII 700 | Atlantis II 700 m water column, red Sea | [1], [2] | 51443487 | 64636 |
|  | ATII 1500 | Atlantis II 1500 m water column, red Sea | [1], [2] | 32542975 | 39190 |
| Marine hydrothermal vents | GB VNT | Guaymas Basin deep-sea hydrothermal vent plume water, Deep Gulf of California | [2], [3], [4], [5] | 10092836 | 12928 |
|  | K VNT | Kueishantao shallow-sea hydrothermal vent, Taiwan | [2], [6] | 4724790 | 4235 |
|  | LC MM | Loki’s Castle deep-sea vent biofilm (microbial mat) | [2], [6] | 13324405 | 11319 |
| Fresh water | RG | River Ganga, Varanasi, India | GCA_004348215.1 | 18532629 | 24721 |
|  | LL | Lansing Lake, Michigan, USA | GCA_009467185.1 | 23646022 | 7443 |
|  | MSUL | MSU3 Lake, Michigan, USA | GCA_009467265.1 | 5647297 | 1981 |
|  | LEN | Lake Erie, Niagara, Canada | GCA_900249105.1 | 15494770 | 15973 |
|  | SWS | The surface of water catchment in Singapore, WC_Site 4c | GCA_900258585.1, [7] | 40691729 | 46218 |
|  | TLB | Taihu Lake water bloom, China | GCA_001515565.1 | 60186787 | 46225 |
|  | WG | Wintergreen Lake, Michigan, USA | GCA_009469485.1 | 8769751 | 1460 |

References

1. Ferreira AJS, Siam R, Setubal JC, Moustafa A, Sayed A, Chambergo FS, et al. Core Microbial Functional Activities in Ocean Environments Revealed by Global Metagenomic Profiling Analyses. PLOS ONE. 2014;9:e97338.

2. Ziko L, Adel M, Malash MN, Siam R. Insights into Red Sea Brine Pool Specialized Metabolism Gene Clusters Encoding Potential Metabolites for Biotechnological Applications and Extremophile Survival. Mar Drugs. 2019;17.

3. Sheik CS, Jain S, Dick GJ. Metabolic flexibility of enigmatic SAR324 revealed through metagenomics and metatranscriptomics. Environ Microbiol. 2014;16:304–17.

4. Baker BJ, Lesniewski RA, Dick GJ. Genome-enabled transcriptomics reveals archaeal populations that drive nitrification in a deep-sea hydrothermal plume. ISME J. 2012;6:2269–79.

5. Lesniewski RA, Jain S, Anantharaman K, Schloss PD, Dick GJ. The metatranscriptome of a deep-sea hydrothermal plume is dominated by water column methanotrophs and lithotrophs. ISME J. 2012;6:2257–68.

6. Tang K, Liu K, Jiao N, Zhang Y, Chen C-TA. Functional metagenomic investigations of microbial communities in a shallow-sea hydrothermal system. PloS One. 2013;8:e72958.

7. Mitchell AL, Scheremetjew M, Denise H, Potter S, Tarkowska A, Qureshi M, et al. EBI Metagenomics in 2017: enriching the analysis of microbial communities, from sequence reads to assemblies. Nucleic Acids Res. 2018;46:D726–35.
